# Dominance from the perspective of gene–gene and gene–chemical interactions

**DOI:** 10.1007/s10709-015-9875-9

**Published:** 2015-11-27

**Authors:** Arkadiusz Gladki, Piotr Zielenkiewicz, Szymon Kaczanowski

**Affiliations:** Bioinformatics Department, Institute of Biochemistry and Biophysics, Polish Academy of Sciences, ul. Pawinskiego 5a, 02-106 Warsaw, Poland; Plant Molecular Biology Department, University of Warsaw, Warsaw, Poland

**Keywords:** Genetic interactions, Gene–chemical interactions, Haploinsufficiency, Genetic dominance, *Saccharomyces cerevisiae*

## Abstract

In this study, we used genetic interaction (GI) and gene–chemical interaction (GCI) data to compare mutations with different dominance phenotypes. Our analysis focused primarily on *Saccharomyces cerevisiae*, where haploinsufficient genes (HI; genes with dominant loss-of-function mutations) were found to be participating in gene expression processes, namely, the translation and regulation of gene transcription. Non-ribosomal HI genes (mainly regulators of gene transcription) were found to have more GIs and GCIs than haplosufficient (HS) genes. Several properties seem to lead to the enrichment of interactions, most notably, the following: importance, pleiotropy, gene expression level and gene expression variation. Importantly, after these properties were appropriately considered in the analysis, the correlation between dominance and GI/GCI degrees was still observed. Strikingly, for the GCIs of heterozygous strains, haploinsufficiency was the only property significantly correlated with the number of GCIs. We found ribosomal HI genes to be depleted in GIs/GCIs. This finding can be explained by their high variation in gene expression under different genetic backgrounds and environmental conditions. We observed the same distributions of GIs among non-ribosomal HI, ribosomal HI and HS genes in three other species: *Schizosaccharomyces pombe*, *Drosophila melanogaster* and *Homo sapiens*. One potentially interesting exception was the lack of significant differences in the degree of GIs between non-ribosomal HI and HS genes in *Schizosaccharomyces pombe*.

**Electronic supplementary material:**

The online version of this article (doi:10.1007/s10709-015-9875-9) contains supplementary material, which is available to authorized users.

## Introduction

Genetic interaction (GI) is a phenomenon in which the effect (fitness) associated with one gene is modified (enhanced or alleviated) by other gene(s). During the past few years, there has been a breakthrough in the field of genetic interactions thanks to the appearance of the SGA (synthetic genetic array) technique. This high-throughput method has facilitated the exploration of synthetic lethal and synthetic sick genetic interactions on a genome-wide scale. With approximately 30 % coverage of double deletions in the *Saccharomyces cerevisiae* genome (Costanzo et al. 2010), it has become possible to better understand the properties of the cellular network of genetic interactions. The degree of connectivity of this network has been shown to be distributed just as in other biological networks, i.e., the majority of genes have few interactions, whereas a small number of genes are highly connected and serve as network hubs. Negative genetic interaction hubs have been shown to have low expression variation, which makes them less prone to ‘epigenetic’ epistatic interactions (Park and Lehner 2013). The essential genes and other genes showing a strong fitness defect in knockout studies have been observed to have generally more genetic interactions (Costanzo et al. 2010). It has also been shown that genes from the same pathway or biological process tend to have similar profiles of genetic interactions (Costanzo et al. 2010). Among the many interesting applications, combining genetic perturbations with multiple chemicals appears to be a promising step forward, especially in medical research (e.g., anticancer drug discovery; Ashworth et al. 2011).

The history of the study of genetic dominance is much older than that of genetic interaction research. Mendel’s studies, which laid the cornerstone of modern genetics, were performed approximately 150 years ago. In crossing two strains of peas, he noticed that a particular variation of a trait (for example, the greenness of the pea) would not appear in the next generation because the effects of a recessive allele were masked by the presence of a dominant one (Mendel 1901).

Since Mendel’s discoveries, dominant and recessive alleles have been studied thoroughly, especially from an evolutionary perspective (Bürger and Bagheri 2008). The argument between the two fathers of population genetics, R. A. Fisher and Sewall Wright, led to a breakthrough in our understanding of the evolution of dominance. Fisher suggested that dominance arose from direct selection to modify the fitness effect of heterozygotes. Sewall Wright (supported by J. B. S. Haldane) suggested that dominance arose as an indirect effect of selection.

Since the time of the argument between Fisher and Wright, our knowledge has increased considerably. The most widely accepted theory, proposed by Kacser and Burns (MCT; Kacser and Burns 1981), is in agreement with Wright’s view. According to MCT, dominance is considered to be the consequence of the kinetic structure of an enzyme network. Although MCT is in opposition to the ‘gene modifier theory’ proposed by Fisher, it has been shown that in some “special cases”, dominance can be shaped directly by natural selection (Tarutani et al. 2010).

It has been shown (comprising one of the key studies confirming the Wright view) that novel recessive mutations usually cause a loss of gene function (Orr 1991). Additionally, the molecular mechanisms causing the dominance of a number of novel mutations have been identified. Wilkie (1994) reviewed the following mechanisms: reduced gene dosage, expression or protein activity (haploinsufficiency; Seidman and Seidman 2002); increased gene dosage (Patel et al. 1992); ectopic or temporally altered mRNA expression (Ruvkun et al. 1991); increased or constitutive protein activity (Mango et al. 1991); dominant negative effects (Herskowitz 1987); altered structural proteins (Sykes 1990); toxic protein alterations (Monplaisir et al. 1986) and new protein functions (Owen et al. 1983).

Haploinsufficiency is the best-studied type of dominance. Haploinsufficient genes have been shown to predominantly encode “transcription factors and other proteins involved in signal transduction and macromolecular complexes” (Birchler and Veitia 2010). Haploinsufficient genes have attracted the attention of medical researchers because mutations in such genes result in many hereditary diseases (e.g., 299 such genes were found in a rigorous search of the published literature and the OMIM database; Dang et al. 2008). Moreover, many haploinsufficient genes have been shown to be connected with cancer (Santarosa and Ashworth 2004). There are more than 100 known tumor suppressor genes (TSG) in humans. In these cases, haploinsufficiency leads to an inability to maintain cells, one of the causes of cancer (Manikandan et al. 2012).

Haploinsufficiency has been most thoroughly studied in yeast. Several high-throughput studies of this matter have been conducted in *Saccharomyces cerevisiae* (Deutschbauer et al. 2005; studies by Oliver’s group, i.e., Delneri et al. 2008; Gutteridge et al. 2010; Pir et al. 2012). Importantly, various experimental techniques for the detection of haploinsufficiency were used in those studies. Moreover, searches for haploinsufficient genes were conducted under various culture media conditions. There was also one high-throughput study conducted in *Schizosaccharomyces pombe* (Baek et al. 2008).

Haploinsufficient genes are also well recognized by the *Drosophila melanogaster* research community. In this species, loss-of-function dominant mutations result in specific, repeatable phenotypes (prolonged development, short and thin bristles, poor fertility and viability) called Minutes. The initial studies of these phenotypes were conducted approximately 90 years ago (Bridges and Morgan 1923). However, additional investigations were needed to show that almost all such mutations occur in cytoplasmic ribosomal genes (Marygold et al. 2007). Currently, these genes are also attracting the attention of medical researchers, as some human mutations affecting ribosomes (ribosomopathies) lead to disorders with specific clinical phenotypes (Narla and Ebert 2010).

In this study, we looked at genetic dominance from the perspective of gene–gene and gene–chemical interactions. We tried to draw general conclusions about relationships between dominance and sensitivity to different intracellular (gene–gene) and extracellular (chemical–gene) perturbations in compliance with commonly accepted MCT theory and the common assumption that selection acts only indirectly on dominance. We made use of current knowledge about the relationship between genetic dominance and the degree of GIs, indicating that dominant genes generally have more genetic interactions than recessive genes (shown for human haploinsufficient genes in a probabilistic functional interaction network (Huang et al. 2010) and for *S. cerevisiae* HI genes (Park and Lehner 2013). We also considered known factors correlating with the GI degree (Koch et al. 2012; Park and Lehner 2013), especially gene importance (assessed by the fitness defect of gene knockout) and gene expression variation. We concentrated our efforts on *Saccharomyces cerevisiae*, the only species for which abundant genome-wide data are currently available.

We may outline the current study as follows. First, we will evaluate differences in the distribution of genetic interactions between dominant and recessive genes in four organisms: *S. cerevisiae*, *S. pombe*, *D. melanogaster* and *Homo sapiens*. Next, we will search for confounding variables (in *S. cerevisiae* only) potentially affecting the relationship between genetic dominance and genetic interactions, and we will test this relationship with confounding variables taken into consideration. Finally, we will reproduce gene–gene analyses (in *S. cerevisiae* only) on gene–chemical data. We will also make the important point that experimental design affects whole-genome-deletion type data in the analyzed yeasts.

## Materials and methods

### Ribosomal genes

Lists of cytoplasmic ribosomal genes for the studied species were obtained from the Ribosomal Protein Gene Database (Nakao et al. 2004).

### Dominance phenotypes

As our primary source, we used the set of haploinsufficient and recessive (haplosufficient) *S. cerevisiae* genes published by Oliver’s group (Pir et al. 2012). The authors demonstrated a condition dependence of haploinsufficiency and haploproficiency. Briefly, they analyzed haploinsufficiency and haploproficiency under rich-medium conditions by conducting competitive fitness profiling of heterozygous yeast deletion strains in a chemostat and a turbidostat. They observed a strong correlation between those two experiments, but there was a considerable difference in the set of HI (haploinsufficient), HS (haplosufficient) and HP (haploproficient) genes. It should be noted that in the cited study, there was no wild type strain (without deletion) control among the mix of deletions. Thus, both the HI and HP gene categories are, most likely, inflated, whereas the number of HS genes is, most likely, underestimated. To counteract these probable biases, we restricted our analysis to genes that had the same pattern in both experiments (e.g., HI set defined as genes found to be HI in both experiments) to remove probable false positives, especially in the case of the HI and HP datasets. In all sets, the ribosomal genes were filtered out.

As a complementary source of *S. cerevisiae* data, we also used two other studies where competitive fitness profiling was conducted in batch cultures for both heterozygous and homozygous deletion strains (Deutschbauer et al. 2005 and Steinmetz et al. 2002). For more details, please see Online Resource 4 (Deutschbauer et al.) and Online Resource 6 (Steinmetz et al.).

We also analyzed dominance phenotypes in three other species: *Schizosaccharomyces pombe, Drosophila melanogaster* and *Homo sapiens* (see Online Resource 1 for more details).

### Genetic interactions

We used the best studied network of *S. cerevisiae*, constructed by Costanzo et al. (2010). They modeled colony size as a multiplicative combination of the mutant fitness, time, and experiment. Then, they compared the fitness of single deletions with double deletions, introducing a genetic interaction score (ε) metric. Costanzo et al. inferred negative genetic interactions in cases where the fitness of double deletions was significantly higher than the additive effects of single deletions (genetic interaction score significantly less than zero). Analogously, Costanzo et al. inferred positive genetic interactions for cases in which genetic interaction scores were significantly greater than zero.

We followed the recommendation of Costanzo et al. for high-throughput studies and used the dataset with a stringent cutoff applied. In that dataset, a negative interaction between two given genes was inferred if the genetic interaction score (ε) was below −0.12 (and the *p* value <0.05). Positive interaction was inferred if ε was >0.16 (and the *p* value <0.05).

As a second dataset in the analysis, we used all other high-throughput studies of genetic interactions (excluding the Costanzo data) conducted to date. We retrieved them from BioGRID (Stark et al. 2006). Similarly to Costanzo et al., we considered BioGRID interactions annotated as phenotypic enhancement, synthetic growth defect and synthetic lethality to be negative genetic interactions. Conversely, BioGRID interactions annotated as phenotypic suppression and synthetic rescue were considered positive.

We also analyzed the distribution of genetic interactions in three other species: *Schizosaccharomyces pombe, Drosophila melanogaster* and *Homo sapiens* (see Online Resource 1 for more details).

### Gene expression variation

We obtained data from Choi and Kim (2009; supplementary materials), who gathered genome-wide data on gene expression variation resulting from stochastic noise, environmental perturbations, genetic perturbations and evolutionary changes.

### Single mutant fitness, multifunctionality and expression level

We obtained the data from Koch et al. (2012; supplementary materials) who gathered genome-wide data for *S. cerevisiae* from various studies.

### Chemogenetic interactions

Chemogenetic interactions for both heterozygous and homozygous collections of *S.cerevisiae* deletion strains were obtained from the study of Hillenmeyer et al. (2008; supplementary materials).

### Statistical methods

In most cases, the properties of the studied sets of genes/alleles did not follow normal distributions. Thus, we applied a nonparametric method, namely, a two-sample permutation test, to evaluate the statistical significance of observed differences between the distributions (two-sided, *p* value 0.05, with 100,000 Monte Carlo replications). Standard errors were generated from 10,000 random permutations and defined as one standard deviation below and above the mean.

All statistical analyses were conducted in R. We used the MASS package (Venables and Ripley 2002) to conduct multiple regression analyses for *S. cerevisiae* and *S. pombe* data. The perm package (Fay and Shih 2012) was used to conduct the two-sample permutation tests.

We chose a negative binomial regression model as our multiple regression model. For the count data, we evaluated four possible regression models (Poisson binomial regression, negative binomial regression, zero-inflated binomial regression, and zero-inflated Poisson regression). Of these, the best-fitting model was the negative binomial regression model.

We analyzed the enrichment of Gene Ontology terms (Ashburner et al. 2000) with Ontologizer (Bauer et al. 2008).

## Results

### Haploinsufficient genes have more genetic interactions than recessive genes

We studied the degree of genetic interactions in *S. cerevisiae* with two datasets: (1) the high-throughput study of Costanzo and (2) all the other HT studies, merged as one dataset. In all cases, cytoplasmic ribosomal genes were treated as a separate group, i.e., they were filtered out from other haploinsufficient genes. For clarity, starting with the next paragraph, we use the term *haploinsufficient genes* (HI) when we discuss haploinsufficient non-ribosomal genes. Analogously, we use the term *haplosufficient genes* (HS) when we discuss haplosufficient non-ribosomal genes.

**Fig. 1:**
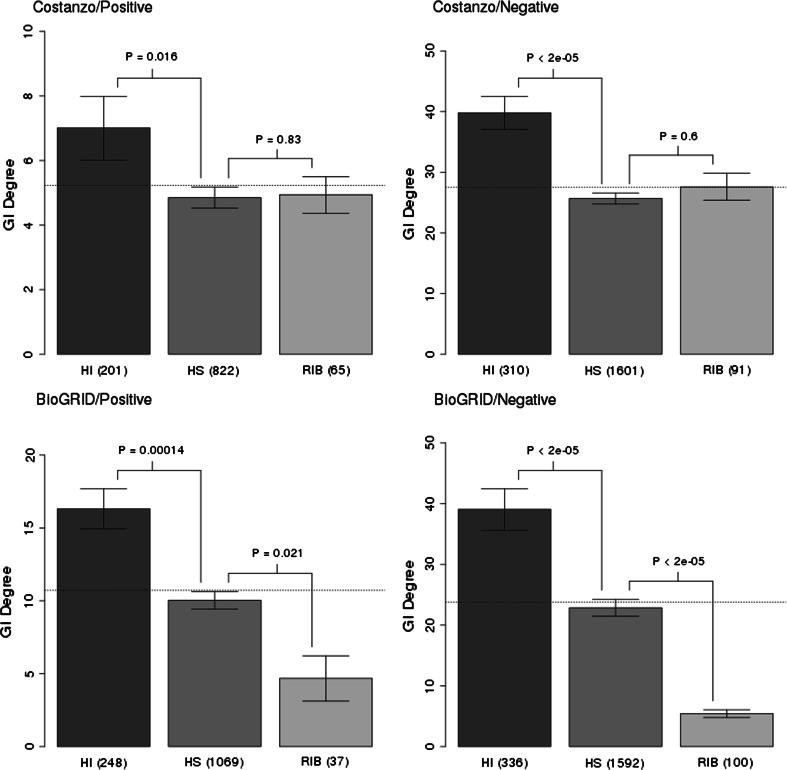
Degree of genetic interactions (positive in the first column, negative in the second) observed for dominant haploinsufficient (in *orange*), recessive (in *blue*) and ribosomal (in *green*) genes in *S. cerevisiae*. Merged high-throughput studies from BioGRID and a single high-throughput study by Costanzo were used. The HI and HS sets were inferred from the Pir et al. study. Haploinsufficient genes have significantly more interactions than recessive ones. Ribosomal genes are depleted in genetic interactions. The means are shown, and the *error bars* represent one standard deviation of the mean over 10,000 bootstrapped samples of the distribution. A two-sample permutation test (two-sided, *p* values are shown above the *error bars*) was used to evaluate the difference between selected sets of genes. The number of genes in selected sets is shown in brackets. The *horizontal dotted line* represents the genome average. *HI*—non-ribosomal haploinsufficient genes, *HS*—haplosufficient (recessive) genes, *RIB*—ribosomal genes. (Color figure online)

We used the Pir et al. study as our primary source of HI and HS genes in *S. cerevisiae*. We found that HI genes had more genetic interactions in both the Costanzo and BioGRID GI sets (Fig. 1). We also found that HS genes had fewer genetic interactions than genes on average (*p* values = 0.067 and 0.4 in case of Costanzo and BioGRID data respectively), while HI genes had significantly more genetic interactions than genes on average (*p* values = 4e−05 in both Costanzo and BioGRID data). These results suggest that the HI and HS sets from the Pir et al. study are representative of the whole genome.

Our analysis distinguished between two main classes of genetic interactions: positive and negative ones. Those classes are usually associated with fundamentally different biological interpretations. Thus, it was not obvious, albeit expected, that the trend would be similar in both cases. Indeed, in both cases, HI genes were observed to have a significantly higher number of both positive and negative genetic interactions in comparison with HS genes (with *p* values = 0.016 and <2e−5 in case of Costanzo data and *p* values = 1.4e−4 and <2e−5 in case of BioGRID data).

*S. cerevisiae* is not the only model organism in which analyses combining the GI network and haploinsufficiency are possible. We conducted similar analyses for *S. pombe*, *D. melanogaster* and *H. sapiens* and observed similar patterns (see Online Resource 1 for more details). One potentially interesting exception was the lack of significant differences in the degree of GIs between HI and HS genes in *S. pombe*. However, those analyses appear to be of limited value because of observed data quality issues, e.g., small GI network in *S. pombe*, network of *H. sapiens* based on co-occurrence data, and set of small scale-studies in the *D. melanogaster* biased towards dominant genes (see Online Resource 1 for more details).

### Dominance significantly correlates with GI degree after taking confounding variables into account

**Fig. 2:**
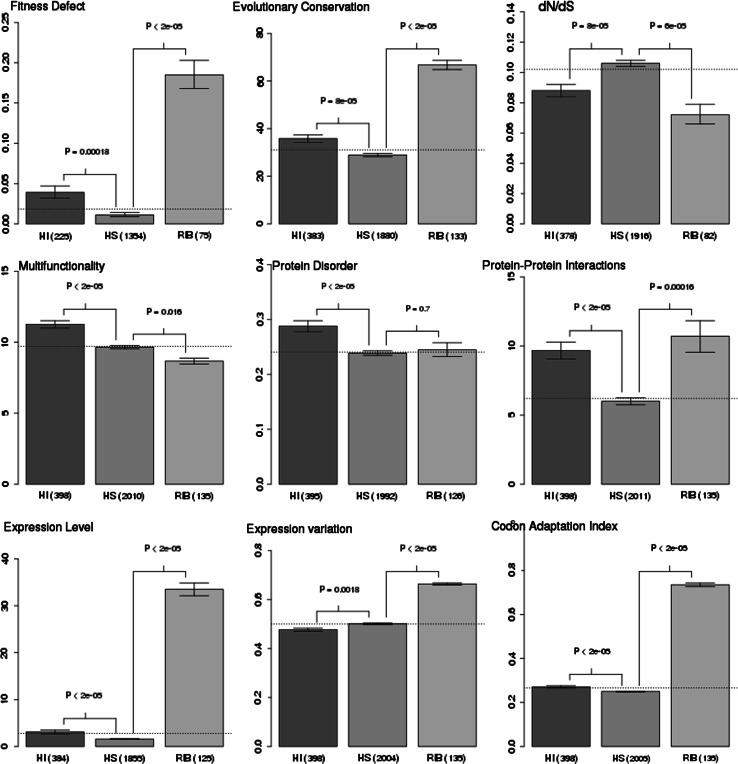
Distribution of selected properties (known to be correlated with GI degree) among three groups of genes: haploinsufficient genes (HI; in *orange*), haplosufficient genes (HS; recessive; in *blue*) and ribosomal genes (in *green*) HI genes (compared with HS genes) are more important genes (stronger single fitness defect), more evolutionarily constrained [higher evolutionarily conservation and lower rate of evolution (dN/dS)], more pleiotropic [i.e., participate in more functions in the cell as indicated by: higher number of Gene Ontology terms (multifunctionality), higher fraction of protein disorder and higher number of protein–protein interactions]. Moreover, HI genes are more highly expressed, have lower variation in gene expression and better optimized gene expression (i.e., codon usage bias as indicated by CAI). Ribosomal genes (in comparison with HS genes and genome average), similar to HI genes, are more important genes, more evolutionarily constrained, and have higher gene expression (one order of magnitude difference). However, (opposite to the case of HI genes) ribosomal genes are less pleiotropic and have a higher variation in gene expression. The HI and HS sets were inferred from the Pir et al. study. Ribosomal genes were filtered out from both HI and HS groups. The means are shown, and the error bars represent one standard deviation of the mean over 10,000 bootstrapped samples of the distribution. A two-sample permutation test (two sided, *p* values are shown above the *error bars*) was used to evaluate the differences between selected sets of genes. The number of genes in selected sets is shown in brackets. The *horizontal dotted line* represents the genome average. *HI*—non-ribosomal haploinsufficient genes, *HS*—non ribosomal haplosufficient (recessive) genes, *RIB*—ribosomal genes. (Color figure online)

Koch et al. (2012) analyzed the correlation of negative GIs with twenty different factors. They conducted the analysis for non-essential genes of *S. cerevisiae* with GI data from the Costanzo study. Koch et al. showed that the negative GI degree correlated with many factors. We checked whether these properties have different distributions in HI and HS genes (Fig. 2). Indeed, we found that HI genes were more important (in terms of a stronger fitness defect of gene knockouts; *p* value = 1.8e−4), more multifunctional (higher level of disorder; *p* value < 2e−5, more protein–protein interactions; *p* value < 2e−5, more Gene Ontology terms describing molecular functions, on average; *p* value < 2e−5) and more evolutionarily constrained (slower evolving genes, i.e., lower dN/dS; *p* value = 8e−5, higher evolutionary conservation; *p* value = 8e−5). We also observed that HI genes have a higher level of gene expression (about twofold; *p* value < 2e−5) and optimized expression (*p* value < 2e−5), as indicated by codon usage bias estimated with CAI and Nc.

**Fig. 3:**
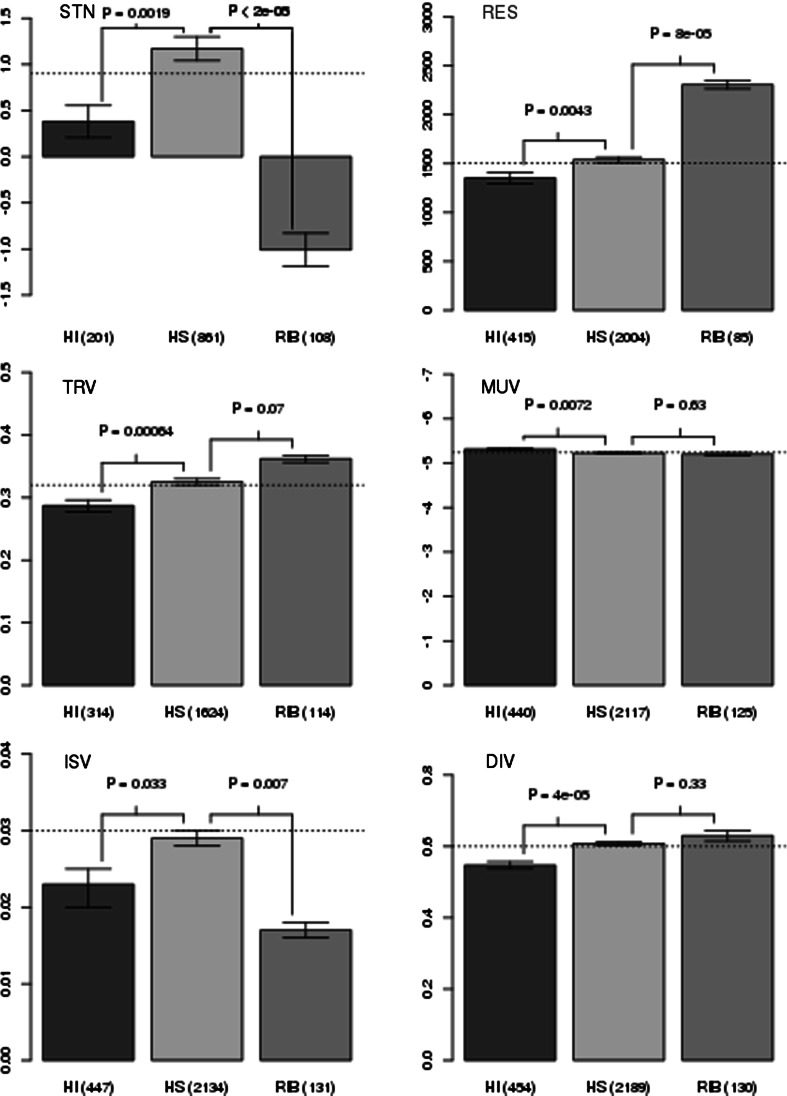
Comparison of distribution of gene expression variation (known to be negatively correlated with GI degree) among three groups of genes: haploinsufficient genes (HI; in *orange*), haplosufficient genes (HS; recessive; in *blue*) and ribosomal genes (in *green*). HI genes have a lower variation in gene expression (than HS genes and genome average) regardless of the source of the variation (stochasticity: STN, environmental perturbations: RES, genetic perturbations: TRV and MUV (reversed Y-axis) or evolution: ISV and DIV). The pattern of gene expression variation is much more complex in the case of ribosomal genes. Ribosomal genes have very low stochastic variation, while the variation originating from environmental and genetic perturbations is surprisingly high. The HI and HS sets were inferred from the Pir et al. study. Ribosomal genes were filtered out from both HI and HS groups. The means are shown, and the *error bars* represent one standard deviation of the mean over 10,000 bootstrapped samples of the distribution. A two-sample permutation test (two-sided, *p* values are shown above the *error bars*) was used to evaluate the differences between selected sets of genes. The number of genes in selected sets is shown in brackets. *Horizontal dotted line* represents the genome average. *HI*—non-ribosomal haploinsufficient genes, *HS*—non ribosomal haplosufficient (recessive) genes, *RIB*—ribosomal genes, *STN*—stochasticity, *RES*—responsiveness, *TRV*—trans variability, *MUV*—mutational variance, *ISV*—interstrain variation, *MUV*—mutational variance. (Color figure online)

Park and Lehner (2013) showed that GI degree is negatively correlated with variation in gene expression regardless of the source of this variation (i.e., stochasticity, genetic perturbations, environmental perturbations, evolution). We found the variation in gene expression to be lower in HI genes in comparison to HS genes and genes on average (Fig. 3). This trend was statistically significant for all four measures and with consideration of the different sources of gene expression variation (classification proposed by Choi and Kim 2009; *p* value = 1.9e−3 in case of stochasticity, p value = 4.3e-3 in case of environmental perturbations, *p* values = 6.4e−4 and 7.2e−3 in case of two measures of genetic perturbations and *p* value = 0.033 and 7e−3 in case of two measures of evolution).

**Fig. 4:**
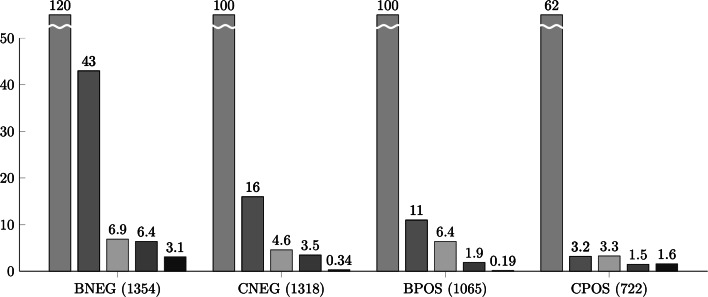
Comparison of effects of selected properties: evolutionary constraints (as single-mutant fitness—in *blue*), multifunctionality (in *red*), genetic dominance (in *beige*), variation in gene-expression (in *grey*) and gene expression level (in *violet*) on GI degree. A negative binomial regression was conducted for each GI network as a function of the selected properties. In each case (except CPOS, probably due to missing data for a large fraction of the genes), dominance significantly affected the GI degree even after taking into account confounding factors (especially single-mutant fitness, multifunctionality and variation in gene expression). The statistical significance of the regression is shown by −log10 (*p* value) on the y axis. The threshold of statistical significance was 1.3 (−log10 of 0.05). The values of single-mutant fitness are rescaled (values orders of magnitude larger than other analyzed properties). The analysis was conducted for the *S. cerevisiae* HI and HS genes identified in Pir et al. study. The numbers of genes analyzed in each GI network are shown in brackets. *BNEG* negative GIs from BioGRID; *CNEG* negative GIs from Costanzo study; *BPOS* positive GIs from BioGRID; *CPOS* negative GIs from Costanzo study. (Color figure online)

We conducted a negative binomial regression analysis to assess how genetic dominance affects the GI distribution and how it is affected by other factors known to be correlated with the GI degree (Fig. 4; see also Online Resource 2). As expected, single mutant fitness correlated most strongly with GI. Other factors also slightly affect the correlation between the GI degree and dominance (see Online Resource 2). Importantly, in the final model dominance was still significantly correlated with the GI degree (Fig. 4; see also Online Resource 3).

### HI genes are more sensitive to chemical perturbations than recessive genes or the genome average

Hillenmeyer et al. (2010) performed more than 1100 chemical genomic assays on the whole-genome set of heterozygous (single allele) and homozygous (double allele) deletion mutants. Thus, they were able to assess the sensitivity of each deletion strain to a plethora of different chemical perturbations by evaluating the level of growth fitness defect.

**Fig. 5:**
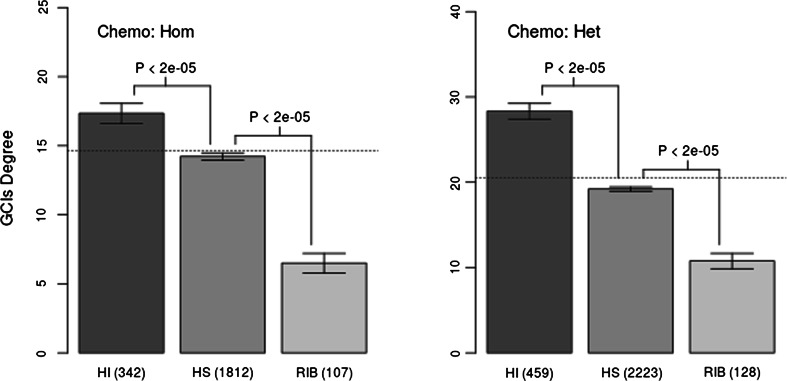
Degree of gene–chemical interactions (for heterozygous deletion strains in the first column and homozygous deletion strains in in the second column) observed for dominant haploinsufficient (in *orange*), recessive (in *blue*) and ribosomal (in *green*) genes in *S. cerevisiae*. A single high-throughput study by Hillenmayer et al. was used. The HI and HS sets were inferred from the Pir et al. study. Haploinsufficient genes have significantly more gene–chemical interactions than recessive ones. Ribosomal genes are depleted in gene–chemical interactions. The means are shown, and the error bars represent one standard deviation of the mean over 10,000 bootstrapped samples of the distribution. A two-sample permutation test (two sided, *p* values are shown above the *error bars*) was used to evaluate the differences between selected sets of genes. The number of genes in selected sets is shown in brackets. The *horizontal dotted line* represents the genome average. *HI*—non-ribosomal haploinsufficient genes, *HS*—haplosufficient (recessive) genes, *RIB*—ribosomal. Chemo: Het: heterozygous chemogenetic network; Chemo Hom: homozygous chemogenetic network. (Color figure online)

**Fig. 6:**
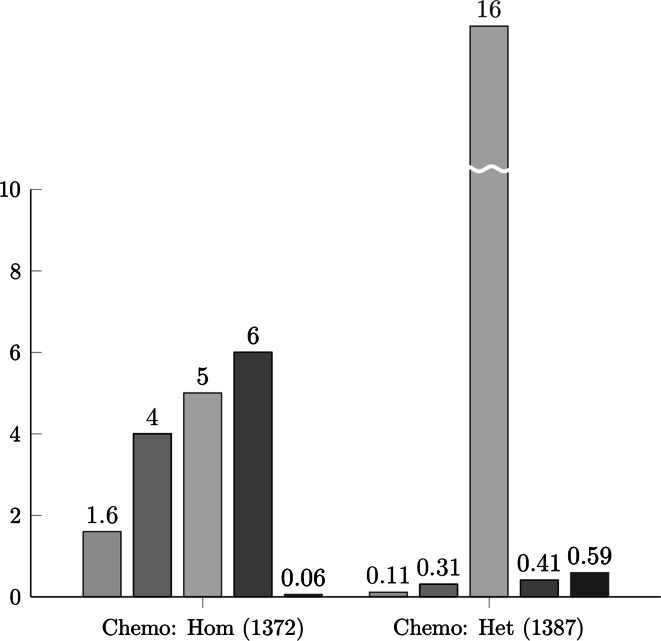
Comparison of effects of selected properties: evolutionary constraints (as single mutant fitness—in *blue*), multifunctionality (in *red*), genetic dominance (in *beige*), variation in gene-expression (in *grey*) and gene expression level (in *violet*) on GCI degree. A negative binomial regression was conducted for each chemogenetic network (built upon a collection of homozygous and heterozygous deletion mutants) as a function of the selected properties. In both homozygous and heterozygous chemogenetic networks, dominance significantly affected the GI degree even after taking into account confounding factors (especially single mutant fitness, multifunctionality and variation in gene expression). The statistical significance of the regression is shown by −log10 (*p* value) on the y axis. The threshold of statistical significance was 1.3 (−log10 of 0.05). The values of single-mutant fitness are rescaled (values orders of magnitude larger than other analyzed properties). The analysis was conducted for the *S. cerevisiae* HI and HS genes identified in Pir et al. study. The numbers of genes analyzed in each GI network are shown in brackets. Chemo: Het: heterozygous chemogenetic network; Chemo Hom: homozygous chemogenetic network. (Color figure online)

We used the aforementioned chemogenetic data to evaluate whether dominant and recessive genes differ in sensitivity to chemical perturbations in both homozygous and heterozygous collections of deletion strains. We observed that dominant genes were significantly more sensitive to chemical perturbations in both heterozygous and homozygous deletion strains (*p* value <2e−5 in both cases; Fig. 5). We reproduced the results with negative binomial regression models (analogous to models in GI degree analyses, with the same confounding factors taken into account; Fig. 6).

In the case of homozygous deletion strains, we observed the same results as in the case of GI analyses, i.e., genes with stronger fitness defects, more pleiotropic genes and genes with lower variation in gene expression were found to be more sensitive to chemical perturbations. However, the correlation between the level of chemical perturbation and fitness growth defects was significantly lower than in the case of the GI degree analyses (approximately 100 orders of magnitude lower, with the *p* value slightly smaller than the significance level, i.e., 0.025). Importantly, after taking all these factors into account, we found that homozygous (double) mutants of dominant genes were more prone to chemical perturbations than homozygous mutants of recessive genes.

Heterozygous deletion mutants of HI genes were found to be strongly sensitive to chemical perturbations relative to recessive genes. Moreover, other factors such as multifunctionality, variation in gene expression, fitness and gene expression level were not found to affect the observed higher sensitiveness of HI mutants to chemical perturbations. It is probable that this finding is the most striking result of our analysis. Note that all previous networks analyzed above (of gene–gene and gene–chemical interactions) were constructed based on the phenotypes (fitness decrease) of homozygous deletion strains. Here, phenotypes of heterozygous deletion strains were evaluated. To our knowledge, this is the only such high-throughput study conducted in *S. cerevisiae*. Moreover, it is probable that this is the most valuable study from the perspective of dominance, as mutations of haploinsufficient genes result from insufficient gene dosage (heterozygous deletion) rather than complete lack of gene expression (homozygous deletion strains).

The results of our analysis of the Hillenmeyer heterozygous dataset indicated that there are probably a large number of novel gene–gene interactions that can be inferred from high-throughput studies of the fitness of heterozygous double deletions. To date, almost all high-throughput genetic interactions have been inferred from homozygous double deletion mutants where there was complete lack of gene expression for two given genes of interest. DAmP (Schuldiner et al. 2005) and temperature sensitive (Ts; Ben-Aroya et al. 2010) deletion mutants are the exceptions. However, such mutants were constructed, in most cases, only for essential genes, which are predominantly recessive genes. Moreover, in the genetic interaction studies conducted to date, DAmP and Ts mutants comprised queries, whereas their baits were always homozygous (double deletion) mutants.

### Ribosomal genes comprise a unique group of HI genes, being depleted in negative and positive GIs as well as GCIs

Cytoplasmic ribosomal genes were analyzed separately, as we assumed, for the following reasons, that cytoplasmic ribosomal genes could bias the results considerably. First, they were considered over studied. Secondly, they are highly important genes (strong fitness defect of gene knockout (Fig. 2; *p* value <2e−5), large fraction of essential genes). We expected, therefore, that they would, most likely, form hubs in the GI network.

The genetic picture of cytoplasmic ribosomal genes turned out to be rather unexpected (Fig. 1). Cytoplasmic ribosomal genes had fewer GIs than non-ribosomal HI genes in the Costanzo dataset (with *p* values = 0.24 and 0.012 for positive and negative GIs respectively). Moreover, in the BioGRID dataset, cytoplasmic ribosomal genes had significantly fewer positive and negative GIs than other analyzed groups, i.e. non-ribosomal HI genes (with *p* values = 0.021 and <2e−5 for positive and negative GIs respectively), recessive genes (with *p* values <2e−5 for both positive and negative GIs).

We observed that ribosomal genes in HT studies conducted with *S. cerevisiae* (except for Costanzo) have been understudied (Table 1; *p* value = 5e−19). Still, this finding explains only to some extent the distribution of genetic interactions for ribosomal genes in yeasts. It also showed the value of a single HT study with high coverage, which is definitely less prone to many biases in comparison with the merged set of genetic interaction, even from HT studies.

**Table 1:** Ribosomal genes are understudied in high-throughput studies of genetic interactions

Species	Observed fraction	Expected fraction	*p* value
*S. cerevisiae*	0.161 (351/21823)	0.259 (150/5800)	5e−19
*S. pombe*	0.117 (42/3600)	0.266 (132/4970)	5e−8

Data were obtained from BioGRID. Only studies with at least 100 genes analyzed were considered to be high-throughput ones (51 such studies in case of *S. cerevisiae*, 4 in case of *S. pombe*). Observed fraction was calculated as the number of times when ribosomal genes were queries or baits in HT studies compared with analogous data for all queries and baits in these studies. The expected fraction was calculated as the number of ribosomal genes compared with the number of all protein-coding genes of given species. The high-throughput study with the highest coverage for *S. cerevisiae* (Costanzo et al.) was filtered out from the analysis. The high-throughput study with the highest coverage for *S. pombe* (Frost et al.) was not deposited in BioGRID at the time of data analysis

We also found that deletion mutants of *S. cerevisiae* ribosomal genes were resistant to different chemical perturbations in the case of both homozygous and heterozygous strains. They were observed to be sensitive to a significantly lower number of chemical species in comparison with deletion mutants of both HS and HI genes (Fig. 5; *p* value <2e−5 in all cases).

## Discussion

### Two classes of haploinsufficient genes have different properties, resulting in opposite positions in gene–gene and gene–chemical networks

The pioneering studies on haploinsufficiency conducted in *Drosophila melanogaster* were connected with Minutes mutations. It was found that almost all such mutations occur in cytoplasmic ribosomal genes (Marygold et al. 2007). The first genome-wide study of haploinsufficiency (Deutschbauer et al. 2005) conducted in *S. cerevisiae* confirmed the dominant loss-of-function phenotype of most ribosomal genes and other translation-related genes. A second genome-wide study of haploinsufficiency in *S. cerevisiae* (Pir et al. 2012) revealed that HI genes often participate in the process of gene expression. Thus, HI genes in *S. cerevisiae* are currently considered to be enriched in transcription and translation-related genes.

We found that both classes of HI genes, in comparison with HS genes, differ in properties connected with gene expression. By definition, HI genes are the ones that are dosage-sensitive. Thus, changes in their transcript level are considered to result in phenotypic changes, which were indeed observed in genome-wide studies of *S. cerevisiae*. Therefore, we would expect HI genes to have a low level of stochastic variation in gene expression compared with HS genes. Indeed, Li et al. (2010) showed that genes with low stochastic noise are enriched in gene-expression-related genes. Moreover, we also observed that HI genes are more highly expressed in comparison to HS genes, which agrees well with the higher fraction of essential genes among them and higher fitness defects of their mutants. Observed differences in gene expression properties seem to explain why direct selection is stronger in the case of HI genes (acting indirectly) and is in agreement with MCT theory as proposed by Kacser and Burns (1981).

Costanzo et al. (2010) showed that the fitness defect of mutants correlates best with the number of genetic interactions. Thus, a strong fitness defect allows us to assume that HI genes will have a high number of GIs. Moreover, ribosomal genes with a very high fitness defect should be hubs in a GI network. Indeed, in agreement with such assumptions, ribosomal genes were found to be hubs in the negative GI network predicted by Koch et al. (2012). Unexpectedly, we found only the non-ribosomal HI genes enriched in GIs and GCIs, whereas ribosomal genes were found depleted in GIs and GCIs. The same pattern (GI analysis only) was observed in *S. pombe*, *D. melanogaster* and *Homo sapiens* (with the exception of *S. pombe*, where the differences in GI degrees among non-ribosomal HI genes and HS genes were insignificant). Although the data for these three species suffer from quality issues (see Online Resource 1 for more details), the aforementioned results furnish additional confirmation for the pattern observed in *S. cerevisiae*.

We asked why there was a bimodal distribution of GIs and GCIs observed between two groups of HI genes. We analyzed the functions of non-ribosomal HI genes with Gene Ontology. It was found that non-ribosomal HI genes are often regulatory genes, especially encoding for regulators of gene expression, with transcription factors forming one of the functional classes being overrepresented. We also found that HI genes are often members of the RNA polymerase complex and other macromolecular complexes such as the histone deacetylase complex and the regulatory subcomplex within the proteasome. Moreover, HI genes were found to be located often either in the Golgi apparatus or the nucleoplasm (see Online Resource 8).

It is expected that transcription factors (TFs) and other regulatory genes will tend to have more genetic interactions than other genes. There are two different, not mutually exclusive, potential explanations for this observation. First, it is well known that regulatory genes tend to have genetic interactions with target genes. The second potential explanation is based on the recent studies on expression noise. These studies have shown that in regulatory networks, the propagation of expression noise is attenuated in the case of TFs, whereas, in the case of their target genes (TGs), the noise is enhanced (Li et al. 2010). This finding was shown to be connected with the synergistic interactions between TFs, in which the noise is buffered. Moreover, such buffering was suggested by Huang et al. (2010) in the human probabilistic functional network, where HI genes were found to have more interaction partners and a greater network proximity to other known HI genes than other genes.

We asked whether our observations suggested that gene expression noise could have an impact on the number of genetic interactions. Such a hypothesis was indicated by a recent study by Park and Lehner (2013), which showed that the number of genetic interactions correlated negatively with gene expression noise. Importantly, they showed that such a correlation was observed not only in the case of stochastic variation in gene expression but also in other contexts of gene expression. In more detail, they found that genes with a high degree of GIs degree also have low gene expression variation among different environmental conditions, in different genetic backgrounds (trans-variability) and in the evolutionary context. The authors hypothesized that genes enriched in GIs determine a higher expression robustness in bakers’ yeast cells, which, in turn, determines phenotypic robustness.

The results of the current study agree with Park and Lehner’s hypothesis. Non-ribosomal HI genes have small gene expression variation in all analyzed contexts. Note that we found the non-ribosomal HI genes of *S. cerevisiae* to be enriched in gene–gene and gene–chemical interactions even when we considered confounding variables (including variation in gene expression). In our opinion, this may be connected with the underestimation of the impact of the analyzed confounding variables or with the lack of other confounding variables correlated with the degree of GI and GCI. Importantly, in case of Hillenmeyer heterozygous dataset (GCIs), only the latter explanation is possible.

The small number of GIs and GCIs in the case of ribosomal genes is also in agreement with Park and Lehner hypothesis. While ribosomal genes represent the functional groups of genes with the lowest variation in stochastic gene expression (in the *S. cerevisiae* genome), they also have high gene expression variation in different environmental conditions and in different genetic backgrounds (when comparing to other analyzed groups: non-ribosomal HI, HS and genome average). Such high variation of gene expression among ribosomal genes in different environmental conditions is well explained by the rate of growth (see Regenberg et al. 2006; Airoldi et al. 2009).

### The datasets obtained from the Deutschbauer et al. and Steinmetz et al. studies do not support the findings obtained with the Pir et al. dataset

Deutschbauer et al. (2005) were the first group to use homozygous and heterozygous deletions to predict haploinsufficient genes in *S. cerevisiae* by searching for genes with significant fitness defects in heterozygous deletions. We repeated our chemogenetic and GI network analyses with the Deutschbauer dataset. Surprisingly, the results based on the Deutschbauer datasets are not in agreement with the results observed for the Pir et al. dataset. Importantly, we did not observe a higher number of gene–gene and gene–chemical interactions among HI genes after controlling for confounding factors (see Online Resource 4 for more details). We found that genes encoding for regulators of gene expression (especially those regulating transcription) were overrepresented among the genes excluded from the Deutschbauer et al. study (because of data quality issues; see Online Resource 5). This finding explains the observed differences between compared studies, as regulators of gene expression were, on the contrary, enriched among non-ribosomal HI genes in the Pir et al. dataset.

Interestingly, in the case of the data of Deutschbauer et al. we also did not observe significant relationship between gene expression variation and GI number, while Park and Lehner (2013) described a lower gene expression variation of HI genes in the same dataset when compared with other genes. The observed disagreement stems from differences in procedures. First, Parker and Lehner did not control for confounding variables, especially fitness defects; we did so. Second, they chose too liberal HS dataset (other genes), for which fitness defects are an order of magnitude higher than for the HI dataset.

Steinmetz et al. (2002) were the first group to provide high-throughput data on homo- and heterozygous deletions in yeast cultured in YPD medium. They evaluated fitness defects of gene deletions to find the genes whose deletions result in significantly different growth rates in fermentable media compared with non-fermentable media. Interestingly, Steinmetz’s data were then intensely discussed with respect to dominance (Phadnis 2005; Delneri et al. 2008; Manna et al. 2012). We used Steinmetz’s experimental data (raw reads from Affymetrix Tag3 library) to predict HI and HS genes in a way analogous to the procedure that we applied to the Deutschbauer et al. data (see Online Resource 6 for detailed description). Similar to the results of the analysis of the Deutschbauer data, and also in the case of Steinmetz’s data, we observed a disagreement with the results derived from the analysis of the Pir et al. data. In addition, in Steinmetz’s dataset we found HI genes to have significantly less gene–gene (network of negative GIs) and gene–chemical (homozygous deletion mutants) interactions before and after controlling for confounding variables (see Online Resource 6 for more details). We found that HS genes in Steinmetz’s dataset were enriched in regulators of the gene expression category (see Online Resource 7). In our opinion, they are misclassified and are, in fact, HI genes, which may explain the observed disagreement between the analyses of data derived from Steinmetz and Pir.

### In the case of the yeast model, the detected haploinsufficiency is affected by the experimental design (culture type)

We compared the studies conducted on *S. cerevisiae* (Deutschbauer et al. and Pir et al.) and found significant differences in experimental design and data quality. We believe that the type of culture (batch culture vs. continuous culture) represents a key difference in this case. It has already been shown that continuous cultures were more reproducible and stable than batch cultures (Knijnenburg et al. 2009), with a significantly lower average intralaboratory coefficient of variation (Piper et al. 2002). Thus, it is not surprising that in the case of Deutschbauer et al., predicted fitness defects had a higher level of variation compared with Pir’s. Moreover, they had to apply a very liberal approach to yield significant results (at least one tag of given gene with significantly different growth rate to be considered haploinsufficient or haploproficient, without multiple hypothesis correction; see Online Resource 4 for more details). This is not the case for the Pir et al. studies, which were conducted in continuous cultures.

Note that besides the experimental design, there were also other differences between the Pir et al. and Deutschbauer et al. studies. For example, Pir et al. chose statistical procedures that were less prone to make incorrect assumptions (those authors used non-parametric tests to calculate p value) and less sensitive to outliers, e.g., robust regression models (please see Online Resource 9 for a detailed comparison of the Deutschbauer et al. and Pir et al. studies). However, in our opinion, other differences did not substantially affect the observed discrepancies between these two studies.

We used Steinmetz’s data (derived from batch cultures as well) to predict HI and HS genes similarly as in the case of the Deutschbauer et al. study. We observed the same data variation and similar pattern of GO enrichments among HI genes (presence of ribosomal genes and absence of regulators of gene expression, especially transcription-related), which furnishes an additional confirmation that experimental design (batch cultures) affected the predicted haploinsufficiency in the Deutschbauer et al. study.

The only high-throughput study addressing haploinsufficiency in *S. pombe* was conducted in batch cultures 5 years ago. In our opinion, a re-analysis of fitness defects of S*. pombe* genes in continuous cultures (e.g., by using a microfluidic microchemostat array; see Nobs and Maerkl 2014) will, most likely, significantly improve our knowledge of haploinsufficiency in S*. pombe*, potentially resulting in the same quality shift as that observed in the case of the Pir et al. study of *S. cerevisiae*. Such an experiment is especially interesting in the light of the predominantly haploid life cycle of fission yeast, making this species a unique eukaryotic model organism.

## Electronic supplementary material

Online Resource 1 Additional methods and results for the analyses conducted for *S. pombe, D. melanogaster* and *H. sapiens* (DOC 243 kb)

Online Resource 2 Estimation and confidence intervals of IRR (incident rate ratios) for independent variables used in negative binomial regression models (genetic dominance, fitness defect, gene expression noise, multifunctionality and level of gene expression) for six analyzed networks (four GI networks and two GCI networks) (XLS 29 kb)

Online Resource 3 Analysis of changes of incident rate ratio (IRR) of genetic dominance in the set of 27 negative binomial regression models used to assess relation between genetic dominance and GI degree and GCI degree (Table) (XLS 25 kb)

Online Resource 4 Additional methods and results for the analyses conducted for *S. cerevisiae* with Deutschbauer et al. dataset (DOC 277 kb)

Online Resource 5 Gene Ontology terms enriched among genes excluded (due to data quality and methodological issues) from the Deutschbauer et al. analysis (XLS 64 kb)

Online Resource 6 Additional methods and results for the analyses conducted for *S. cerevisiae* with the Steinmetz et al. dataset (DOC 222 kb)

Online Resource 7 Additional table with a list of HI and HS genes predicted with Steinmetz dataset and Gene Ontology terms enriched among these genes (XLS 161 kb)

Online Resource 8 Gene Ontology terms enriched among non-ribosomal HI genes identified in Pir et al. study (XLS 91 kb)

Online Resource 9 Additional table with comparison of experimental and significant differences in two key studies of haploinsufficiency conducted in *S. cerevisiae*: Deutschbauer et al. and Pir et al. (XLS 29 kb)
