# Supplementary material for: Dominance from the perspective of gene–gene and gene–chemical interactions
Source: Genetica. 2015 Nov 27;144:23–36. doi: 10.1007/s10709-015-9875-9 (PMC4748009; doi:10.1007/s10709-015-9875-9)
Supplement: Supplementary file 1 — Additional methods and results for the analyses conducted for S. pombe, D. melanogaster and H. sapiens (DOC 243 kb) [file 10709_2015_9875_MOESM1_ESM.doc]

**Materials and method**

***Dominance phenotypes***

*Homo sapiens*

We used the manually curated data on Mendelian disease genes (Blekhman et al. 2008) to locate genes with dominant and recessive mutations. We considered a gene to be dominant if there was at least one known disease caused by only dominant mutations of a given gene. We considered a gene to be recessive if all its disease-causing mutations were found to be recessive. We also used a comprehensive list of haploinsufficient genes extracted by rigorous text-searching and database-mining strategy (Dang et al. 2008). We generated a list of dominant genes with gain-of-function mutations by filtering out haploinsufficient genes from the list of all dominant genes found. Finally, we filtered out ribosomal genes from all the three sets.

*Drosophila melanogaster*

Data were obtained from FlyBase (Tweedie et al. 2009). We extracted the information from the XML file (Fbal.xml; version FB 2011_09; see Table A1 for more details about selected fields).

First, we evaluated the dominance phenotypes of fruit fly alleles. We assumed that the dominance of each analyzed allele refers to or is the same in crosses with the wild-type allele. Different phenotypes were often studied for the same allele. We considered each phenotype of a given allele as a separate intragenic interaction. We assumed that a given allele was recessive if at least one recessive phenotype and no dominant phenotypes were detected. An analogous rule was used in the case of dominant alleles. We used Muller’s morphs classification of mutations (Wilkie 1994) to define two groups of dominant alleles: the ones with loss-of-function phenotype (with Muller’s amorphic and hypomorphic phenotypes) and gain-of-function phenotype (the ones with neomorphic, antimorphic and hypermorphic Muller’s phenotypes). We assumed that, in most cases, loss-of-function dominant alleles were mutations in haploinsufficient genes. Groups of gain-of-function alleles were considered to be occurring in dominant genes with several different mechanisms (negative effects, toxic protein alteration, and new protein functions). Alleles with dominant visible and recessive lethal phenotypes (haplolethal) are a specific type of mutations in haploinsufficient genes and were also added to the list of loss-of-function alleles. Finally, we filtered out ribosomal alleles from all the three sets.

We used the alleles with a known dominance phenotype to predict dominant and recessive genes. We considered genes to be haploinsufficient if there was at least one allele with loss-of-function dominant phenotype. We treated the gene as dominant with any gain-of-function mechanism if there were known allele(s) with gain-of-function dominant phenotypes but no loss-of-function dominant phenotypes. Recessive genes were represented by genes with only recessive mutations. Finally, we filtered out ribosomal genes from all three sets.

*Schizosaccharomyces pombe*

We used the lists of haploinsufficient (HI) and recessive (haplosufficient; HS) genes that were identified by Baek et al. 2008 (genome-wide growth fitness profiling). We used the HI, HS and HP (haploproficiency) classification published by the above authors as our primary source. The classification was inferred from both fitness and statistical criteria (fitness < 0.98 and at least one tag for a given gene statistically significant, i.e., p-value < 0.05).

We also generated a secondary set based only on statistical data, similarly to the procedure applied by Pir et al. (2012) for haploinsufficiency analyzed in *S. cerevisiae*, i.e., we applied a multiple testing correction for p-values (with FDR; Benjamini-Hochberg correction). Genes with both tags significant (after correction) were assigned as HI and HP. Genes with both tags insignificant were assigned as HS (recessive). After such corrections, most haplosufficient genes had a fitness between 0.991 and 1.011 (5-th and 95-th centile, respectively). Most HI genes had fitness between 0.915 and 0.980 (5-th and 95-th centile, respectively). Most HP genes had fitness between (1.011 and 1.051 (5-th and 95-th centile, respectively).

In both sets, ribosomal genes were filtered out. We were unable to find or predict a set of dominant genes with gain-of-function mutations.

***Genetic interactions***

In most cases, we restricted our analysis only to genes/alleles with at least one genetic interaction (analysis for *Drosophila melanogaster* constitutes an exception). Different methods were used to detect genetic interactions in the studies species. However, in all cases, genetic interaction was assumed when mutation in one gene/allele affected (enhanced or alleviated) the phenotype of another gene/allele. All duplicated interactions were removed when positive and negative interactions were observed between a given pair of genes (1), when a given interaction was observed in both combinations query-bait and bait-query (2) or when the same interaction was detected in few studies (3). Thus, we defined the GI degree as the number of unique genetic interactions of a given gene.

*Homo sapiens*

We used data from a genome-wide study in which genetic interactions were inferred from hybrid radiation genotypes (Lin et al. 2010)*.* The study used radiation hybrid panels to compare co-occurrence of various genomic fragments.

In the first step, the authors calculated the co-occurrence among all genetic markers (mutations) mapped on human genome. Genetic interaction between any two genetic markers was predicted if these markers were co-retained (positive genetic interaction) or co-lost (negative genetic interaction) in the analyzed genomic fragments more often than expected by chance.

In the next step, Lin et al. mapped genetic interactions of markers into genetic interaction of genes. The authors predicted genetic interaction between two genes if at least one pair of markers located within these genes or their proximity was found to be interacting in the previous step.

In the final step, the authors filtered out interactions between genes located in proximity (below 1 MB) as such DNA fragments of such genes are more often co-retained in radiation hybrid panels than expected by chance. More details on the statistical approach can be found in their publication (Lin et al. 2010).

Experimental GI studies from BIOGRID comprised the second study (1700 interactions). We merged them with two high-throughput experimental studies detecting GIs in humans (Roguev et al. 2013, Laufer et al. 2013). In both cases, data on interacting gene pairs was retrieved from supplementary materials (500 interactions in the first study and 2500 in the latter).

*Drosophila melanogaster*

We used FlyBase to prepare a genetic interaction network for *Drosophila melanogaster*. The information was extracted from the XML file (Fbal.xml; version FB 2011_09; see also TABLE A1).

First, we analyzed the allelic interactions. Our analysis included only one-to-one interactions (i.e., exactly one allele was designated as an enhancer/suppressor of the second one being suppressed/enhanced). Then, we filtered the network down to interactions of alleles with at least one known phenotype and a known chromosomal localization. With this network, we calculated the number of genetic interactions for each allele. A genetic interaction between the allele of interest and a given gene was assumed if at least one allele of a given gene interacted with the allele of interest.

We used allelic interactions to infer genetic interactions. We predicted genetic interaction between two genes if there was an interaction between any alleles of those genes.

*Schizosaccharomyces pombe*

We used the best-studied network of genetic interactions constructed by Frost and co-workers (2012) as our primary source of genetic interactions. We received the data from the authors, with detailed information regarding the threshold for positive and negative interactions.

High-throughput studies of genetic interactions (Frost’s data were not available at the time of our analysis) comprised the second source of genetic interactions (BioGRID, version 3.2.95). The same rules as in the case of *S. cerevisiae* were applied to obtain positive and negative genetic interactions.

***Genetic interaction bias in Drosophila melanogaster***

In the case of *Drosophila melanogaster*, our data consisted of many small-scale studies maintained and updated regularly the by FlyBase consortium. Such data were shown to be more prone to biases in comparison with high-throughput data (Dickerson et al. 2010). This bias occurs because some genes are more interesting to the scientific community and are, thus, expected to have a higher fraction of detected genetic interactions.

To exclude the impact of such bias, we applied the method proposed by Dickerson et al. 2010 (a detailed description of the procedure can be found in their manuscript). We assumed that genes described in numerous scientific publications are more interesting to the scientific community. Hence, we compared the number of genetic interactions of two given classes of genes with the same level of scientific interest (same distribution of publication count). In detail, we applied the rejection sampling algorithm, also known as the accept-reject algorithm. We pseudo-randomized one set of genes (10,000 times) in such a way that their distribution of publication count was the same as for the second set of genes (which is not the case with the random sampling approach). Then, we checked whether there was a statistically significant difference between the number of genetic interactions in the pseudo-randomized set (first group of genes) and the second sets of genes. We performed the analysis using prepared R scripts.

The key information on the number of publications on *D. melanogaster* genes and alleles was obtained by querying the Chado database available in FlyBase. The distribution of publication count was prepared for four sets of alleles (ribosomal, dominant loss-of-function, dominant gain-of-function and recessive alleles) and for analogous sets of genes.

***Statistical methods***

In most cases, the properties of the studied sets of genes/alleles did not follow a normal distribution. Thus, we applied a nonparametric method, a two-sample permutation test, to evaluate the statistical significance of observed differences in distributions (two sided, p-value 0.05, with 10,000 Monte Carlo replications). Standard errors were generated by 10,000 random permutations and defined as one standard deviation below and above the mean.

All statistical analyses were conducted in R. We used the MASS package to conduct multiple regression analyses for *S. pombe* data. The perm package (Fay and Shih 2012) was used to conduct two-sample permutation tests.

We chose the negative binomial regression model as our multiple regression model. The Negative binomial regression model fitted best among the four models for count data analyzed by us (these models were the Poisson binomial regression, negative binomial regression, zero-inflated binomial regression, and zero-inflated Poisson regression models).

**Results**

***S. pombe HI and HS genes have the same distribution of GIs and fitness as the genome average***

*S. pombe* results are an interesting case in the context of the results obtained for the other three species. No difference was observed between the genetic interaction degree of the HI and HS genes (Fig. 2-4). We reproduced the analysis using two different sets of genetic interactions (merged HT studies from BioGRID and a study by Frost et al. (2012) - the one with the highest coverage of the *S. pombe* genome). We also applied an alternative procedure in determining HI and HS genes from the only study conducted so far for *S. pombe* (to obtain analogous datasets as Olivier’s group for the *S. cerevisiae* case, i.e., using only statistical criteria; see Methods for more details). In all cases, considerably small, insignificant differences were observed (see Fig. A1-A2). As in the case of *S. cerevisiae*, *S. pombe* HI genes were found to be more evolutionarily constrained (higher evolutionary conservation, lower evolutionary rate (lower dN/dS)) and more pleiotropic (more GO terms, more PPI interactions, higher disorder; Fig. A3-A4). However, in most cases, the differences were only slightly significant (p-value near the 0.05 threshold). Moreover, no differences were observed when comparing the key properties affecting the GI degree: single-mutant fitness and variation in gene expression as well as other properties connected with gene expression (genes’ expression level, codon usage bias (as indicated by Nc and CAI)).

***The distribution of GIs in case of gain-of-function dominant genes is ambiguous***

We found that the distribution of GI degree in other dominant genes (with different gain-of-function mechanisms) is ambiguous (see Fig. A5). In this case, data were available only for *Homo sapiens* and *Drosophila melanogaster*. While we observed a higher number for the GI degree in the case of *D. melanogaster*, no significant difference was observed between the human HI and HS genes. On the basis of this finding, it is difficult to determine whether the properties shown for haploinsufficient genes can be extrapolated to all dominant genes (at least in predominantly diploid species) regardless of the type of dominance.

***The predicted human network of genetic interactions has unique properties in comparison with SGA networks***

In the case of the human network of negative genetic interactions HI genes were found to have more negative genetic interactions in comparison with recessive genes as well as to genes on average, but this trend was not as highly statistically significant as in the case of positive genetic interactions (see Fig. 3-4). The unique properties of the human network of genetic interactions may provide an explanation for the observed pattern. This network was predicted by examining the co-retention of gene markers in hybrid radiation panels (RH). The authors assumed that positive genetic interaction can be predicted if markers of two genes co-occur in hybrid radiation panels more often than expected (and this finding cannot be explained by their genomic proximity). Analogously, the authors predicted negative genetic interactions if the markers co-occur significantly less than expected by chance (for more details see Methods as well as Lin et al 2010). The authors showed that the probability of detection of positive and negative interactions was the same in their methodology. Surprisingly, they predicted primarily positive interactions (99, 96% interactions, i.e., over seven million positive interactions compared to approximately 3000 negative interactions). Thus, in the network of negative interactions there are very few interactions at all (85% of genes with no negative interactions at all, 0.33 negative interactions per gene on average). We speculate that this may be the reason for the less spectacular differences in negative GIs between HI and HS genes in comparison with the positive GIs. Moreover, such a low negative/positive GI ratio remains in strong contrast to the ratios observed in case of SGA networks in baker’s and fission yeasts. In these yeasts ,negative GIs were observed as often (same order of magnitude) as positive ones (e.g., in the Costanzo network for *S. cerevisiae*, the ratio is 2:1).

Fortunately, new high-throughput methods for the detection of GI in humans have just appeared (Laufer et al., Roguev et al.). Thus, in the near future it will be possible to use experimental data to reproduce the analysis of the human GI degree distribution and its correlation with genetic dominance. There are currently approximately 4100 unique genetic interactions for approximately 1700 human genes in view of the data from BioGRID and two recent high-throughput studies (Laufer et al. 2013 and Roguev et al. 2013). Using this small set of experimental data, we reproduced the analysis and observed the same trend as in the case of human network of predicted genetic interaction (Fig. A6).

**References**

Blekhman R et al. (2008) Natural selection on genes that underlie human disease susceptibility Curr Biol 18:883-889 doi:S0960-9822(08)00601-5

Baek ST et al. (2008) Genome-wide identification of haploinsufficiency in fission yeast J Microbiol Biotechnol 18:1059-1063 doi:7429

Dang VT, Kassahn KS, Marcos AE, Ragan MA (2008) Identification of human haploinsufficient genes and their genomic proximity to segmental duplications Eur J Hum Genet 16:1350-1357 doi:ejhg2008111

Dickerson JE, Pinney JW, Robertson DL (2010) The biological context of HIV-1 host interactions reveals subtle insights into a system hijack BMC Syst Biol 4:80 doi:10.1186/1752-0509-4-80

Fay MP, Shih JH (2012) Weighted logrank tests for interval censored data when assessment times depend on treatment Stat Med 31:3760-3772 doi:10.1002/sim.5447

Frost A et al. (2012) Functional repurposing revealed by comparing S. pombe and S. cerevisiae genetic interactions Cell 149:1339-1352 doi:10.1016/j.cell.2012.04.028 S0092-8674(12)00573-9

Laufer C, Fischer B, Billmann M, Huber W, Boutros M (2013) Mapping genetic interactions in human cancer cells with RNAi and multiparametric phenotyping Nat Methods 10:427-431 doi:10.1038/nmeth.2436

Lin A, Wang RT, Ahn S, Park CC, Smith DJ (2010) A genome-wide map of human genetic interactions inferred from radiation hybrid genotypes Genome Res 20:1122-1132 doi:gr.104216.109

Pir P, Gutteridge A, Wu J, Rash B, Kell DB, Zhang N, Oliver SG (2012) The genetic control of growth rate: a systems biology study in yeast BMC Syst Biol 6:4 doi:10.1186/1752-0509-6-4

Roguev A et al. (2013) Quantitative genetic-interaction mapping in mammalian cells Nat Methods 10:432-437 doi:10.1038/nmeth.2398

Tweedie S et al. (2009) FlyBase: enhancing Drosophila Gene Ontology annotations Nucleic Acids Res 37:D555-559 doi:10.1093/nar/gkn788

Wilkie AO (1994) The molecular basis of genetic dominance J Med Genet 31:89-98

**Figures and Tables**

1. **Fig. A1** Degree of genetic interactions (positive in the first column, negative in the second) observed for dominant haploinsufficient (in orange), recessive (in blue) and ribosomal (in green) genes in *S. pombe*. Merged high-throughput studies from BioGRID and single high-throughput study by Frost were used. HI and HS sets were inferred from Kim et al. study. Haploinsufficient and recessive genes have the GI degree on the level of genome average. Ribosomal genes are understudied and depleted in genetic interactions. Means are shown and error bars represent one standard deviation of the mean over 10000 bootstrapped samples of the distribution. Two-sample permutation test (two sided, p-values are shown above the error bars) was used to evaluate the difference between selected sets of genes. Number of genes in selected sets is shown in brackets. Horizontal dotted line represents the genome average. Abbreviations: HI – non-ribosomal haploinsufficient genes, HS – haplosufficient (recessive) genes, RIB – ribosomal


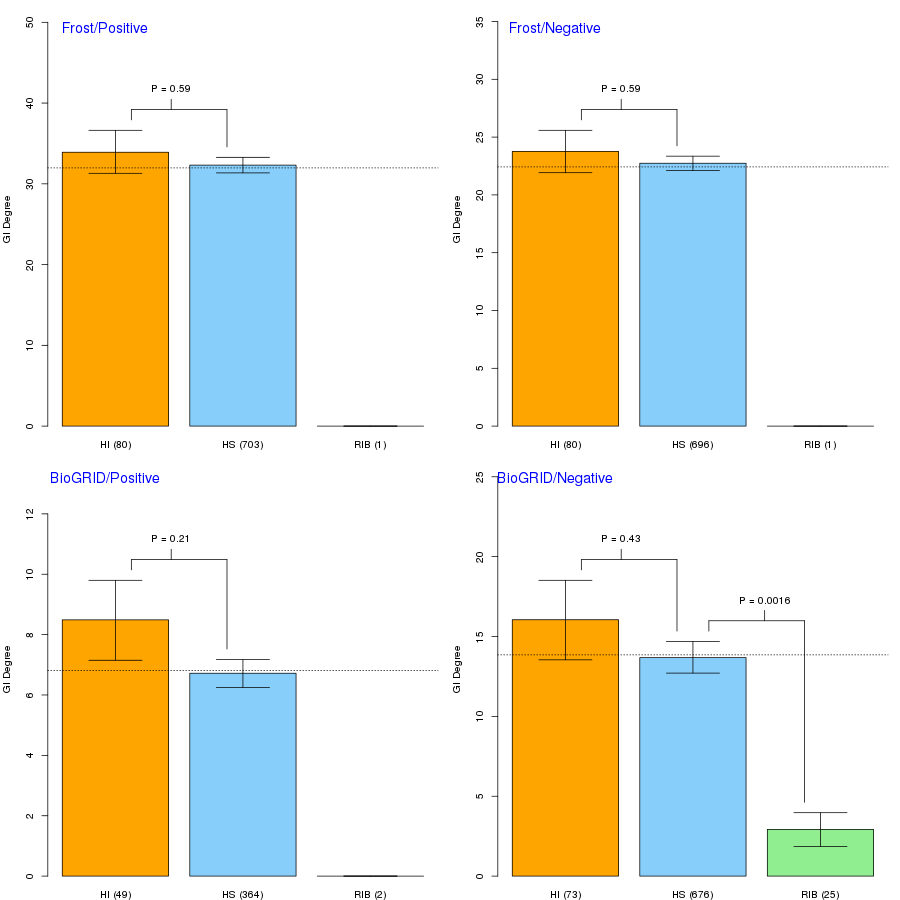


**Fig. A2** Degree of genetic interactions (positive in the first column, negative in the second) observed for dominant haploinsufficient (in orange), recessive (in blue) and ribosomal (in green) genes in *S. pombe*. Merged high-throughput studies from BioGRID and single high-throughput study by Frost were used. HI and HS sets were inferred from Kim et al. study and assigned using only statistical data (with BH correction of ANCOVA p-values). Haploinsufficient and recessive genes have the GI degree on the level of genome average. Ribosomal genes are understudied and depleted in genetic interactions. Means are shown and error bars represent one standard deviation of the mean over 10000 bootstrapped samples of the distribution. Two-sample permutation test (two sided, p-values are shown above the error bars) was used to evaluate the difference between selected sets of genes. Number of genes in selected sets is shown in brackets. Horizontal dotted line represents the genome average. Abbreviations: HI – non-ribosomal haploinsufficient genes, HS – haplosufficient (recessive) genes, RIB – ribosomal


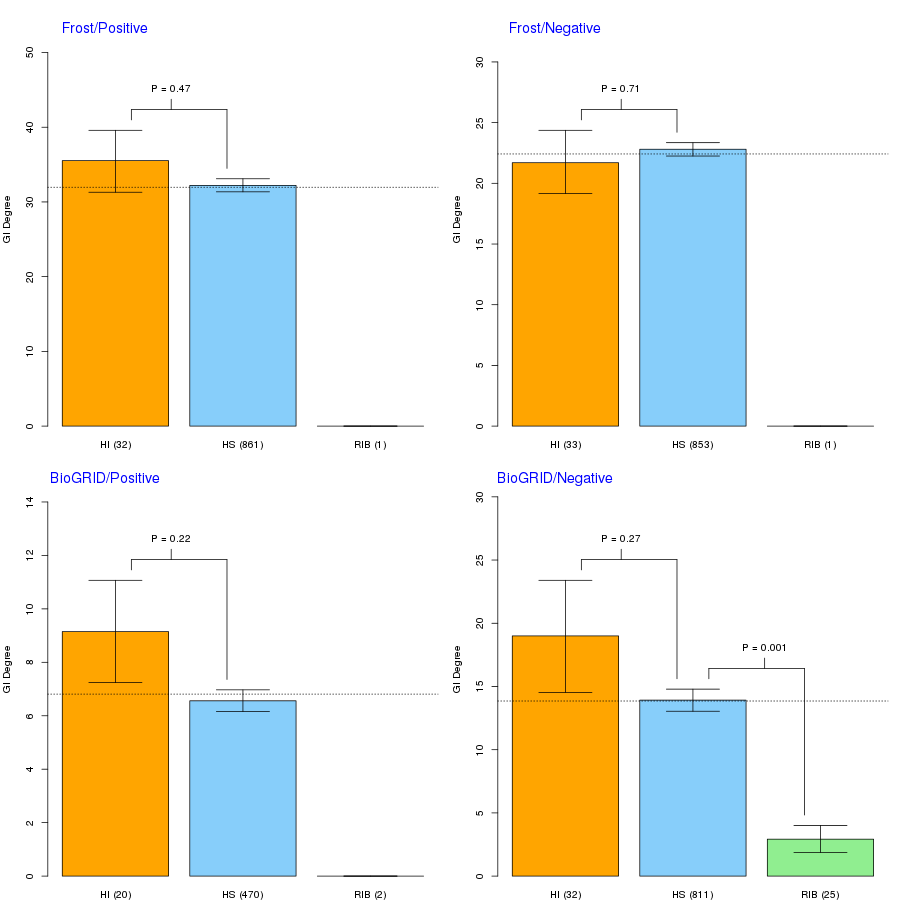


**Fig. A3** Distribution of selected properties (known to be correlated with GI degree) among three groups of *S. pombe* genes: haploinsufficient genes (in orange), haplosufficient genes (recessive; in blue) and ribosomal genes (in green). HI genes (comparing to HS genes) are more evolutionary constrained (higher evolutionary conservation, more pleiotropic (i.e. participate in more functions in the cell as indicated by: higher number of Gene Ontology terms (multifunctionality) and higher number of protein-protein interactions). Opposite to the picture observed in *S. cerevisiae*, HI and HS genes of *S. pombe* have same importance level (comparable single fitness defect), similar level of gene expression, variation in gene expression and codon usage bias (as indicated by CAI). Ribosomal genes as in case of *S. cerevisiae* are more important genes than HI and HS genes, are more evolutionarily constrained, have higher gene expression (one order of magnitude difference). HI and HS sets were inferred from Kim et al. study. Ribosomal genes were filtered out from both HI and HS groups. Means are shown and error bars represent one standard deviation of the mean over 10000 bootstrapped samples of the distribution. Two-sample permutation test (two sided, p-values are shown above the error bars) was used to evaluate the difference between selected sets of genes. Number of genes in selected sets is shown in brackets. Horizontal dotted line represents the genome average. Abbreviations: HI – non-ribosomal haploinsufficient genes, HS – non ribosomal haplosufficient (recessive) genes, RIB – ribosomal genes


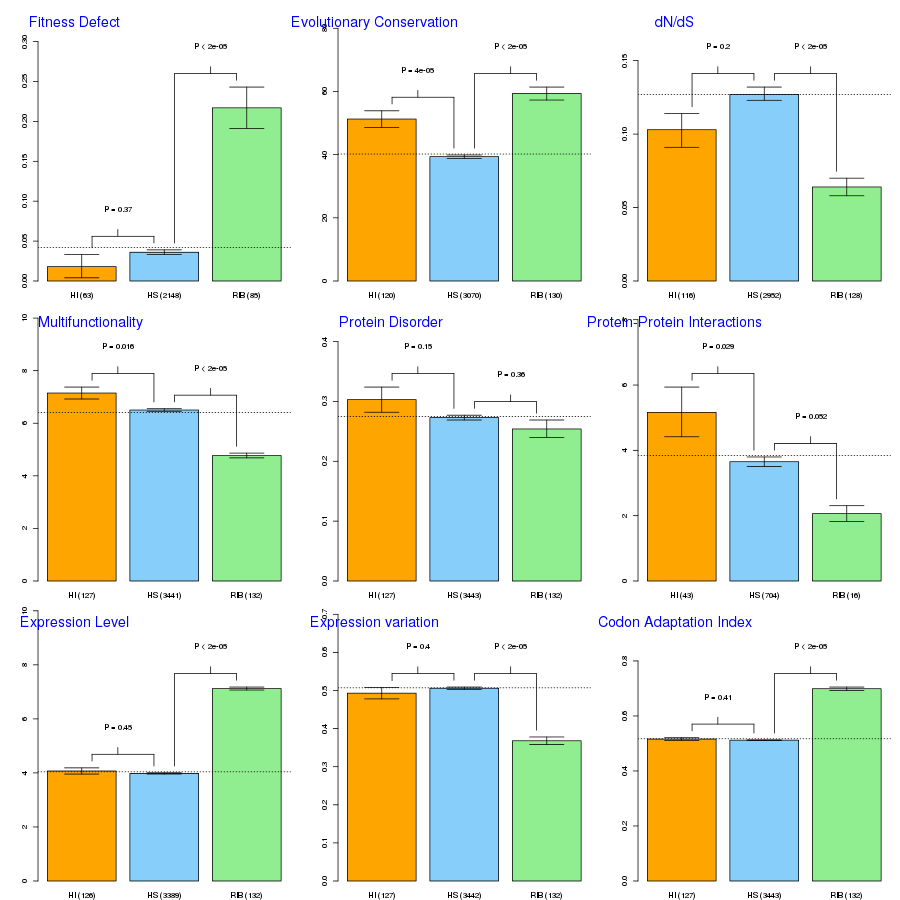


**Fig. A4** Comparison of effects of selected properties: evolutionary constraints (as single mutant fitness – in blue), multifunctionality (in red), genetic dominance (in beige), variation in gene-expression (in grey) and level of gene expression (in violet) on GI degree. Negative binomial regression was carried out for each GI network as a function of selected properties. In each case no correlation between dominance and GI degree is observed when taking into account confounding factors (especially single mutant fitness, multifunctionality and variation in gene expression). The statistical significance of regression is shown by -log10 (Pvalue) on the y axis. The threshold of statistical significance is 1.3 (-log10 of 0.05). Analysis conducted for *S. pombe* genes. Numbers of genes analyzed in each GI network are shown in brackets. Abbreviations: BNEG: negative GIs from BioGRID; FNEG: negative GIs from Frost study; BPOS: positive GIs from BioGRID; FPOS: negative GIs from Frost study. **
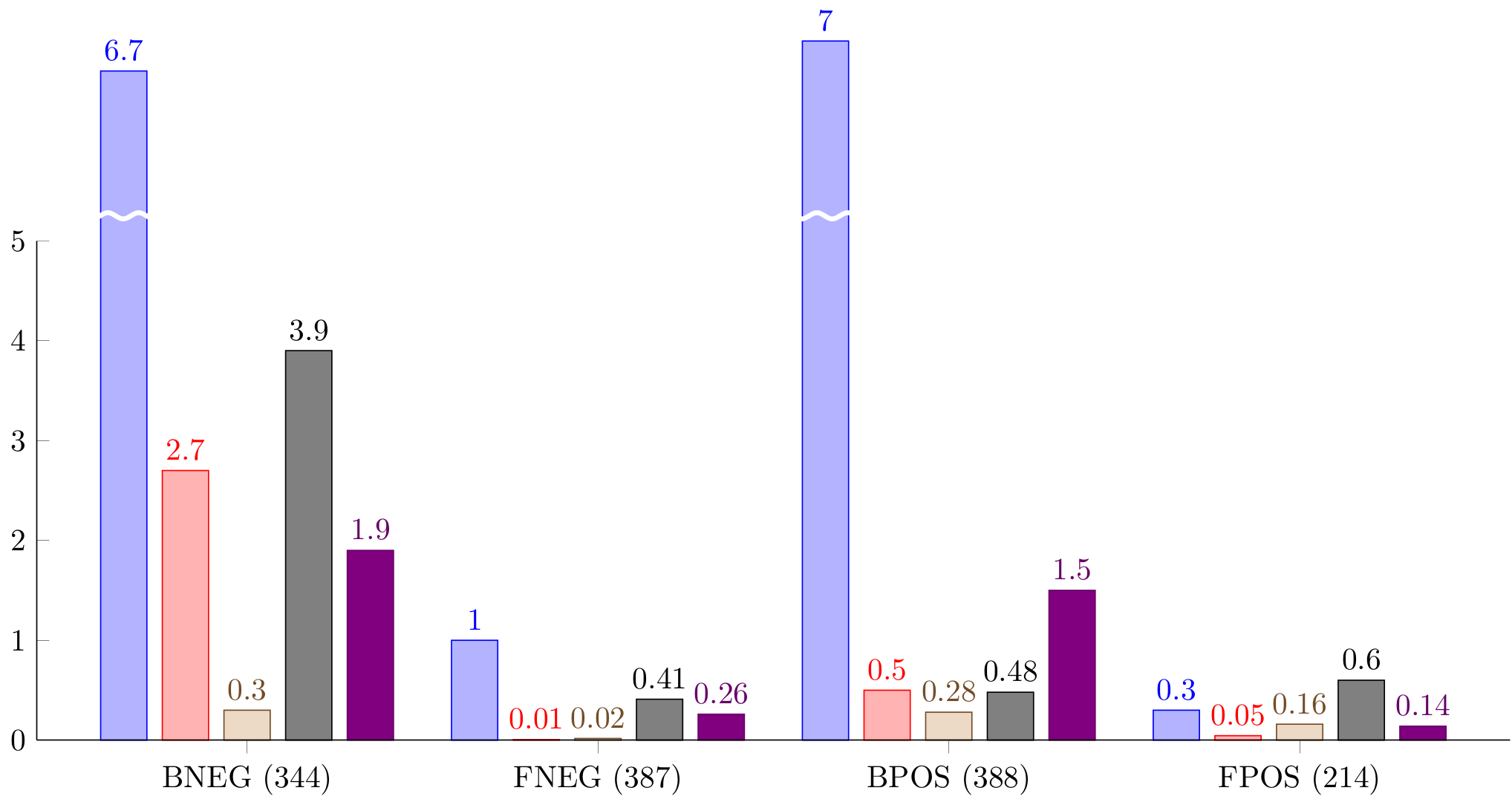
**

**Fig. A5** The distribution of genetic interactions for dominant gain-of-function (in orange) and
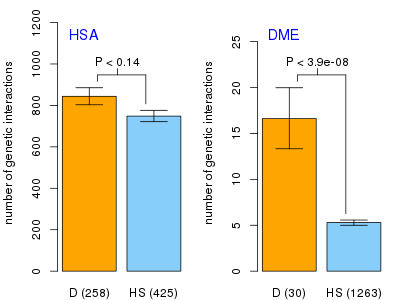
recessive (in blue) genes in human and fruit fly. Dominant gain-of-function genes have more genetic interactions then recessive genes. Means are shown and error bars represent on

e standard deviation of the mean over 10000 bootstrapped samples of the distribution. Two-sample permutation test (two sided, p-values are shown above the error bars) was used to evaluate the difference between selected sets of genes. Number of genes in selected sets is shown in brackets. Abbreviations: HSA – *Homo sapiens*, DME – *Drosophila melanogaster*, D – dominant genes, HS – haplosufficient (recessive) genes


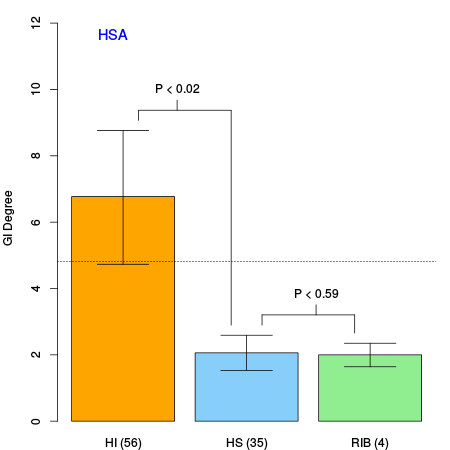
**Fig. A6** Degree of genetic interactions (all - positive and negative) observed for dominant haploinsufficient (in orange), recessive (in blue) and ribosomal (in green) genes in humans. Experimental data from BioGRID as well as from two high-throughput studies (Roguev et al. 2013 and Laufer et al. 2013) were used. Haploinsufficient genes have more GI than recessive genes and compared to genome average. Ribosomal genes are depleted in genetic interactions Means are shown and error bars represent one standard deviation of the mean over 10000 bootstrapped samples of the distribution. Two-sample permutation test (two sided, p-values are shown above the error bars) was used to evaluate the difference between selected sets of genes. Number of genes in selected sets is shown in brackets. Horizontal dotted line represents the genome average. Abbreviations: HI – non-ribosomal haploinsufficient genes, HS – haplosufficient (recessive) genes, RIB – ribosomal

1. **Table A1** XML fields from which some details about fruit fly alleles were extracted.

| Data type | XML Field(s) | Example(s) |
| --- | --- | --- |
| Enhancing interactions (intergenic interactions) | derived_enhancer_class derived_enhancer_manifest derived_enhanceable_class derived_enhanceable_manifest | FBal0189927 is an enhancer of FBal0017976 |
| Suppressing interactions (intergenic interactions) | derived_suppressor_class derived_suppressor_manifest derived_suppressible_class derived_suppressible_manifest | FBal0049166 is a suppressor of FBal0050252 |
| Dominance property (intragenic interaction) | derived_pheno_class derived_pheno_manifest | Dominant, recessive, wild-type |
| Chromosomal localization | Related feature (location) | X, 2L, 4, Y |
| Phenotype | derived_pheno_class derived_pheno_manifest | lethal, viable, stress response defective |
| Muller classification | derived_allele_class | Amorph, neomorph |
| Gene Id | Related feature (alleleof) | FBgn0084606 |
