# Supplementary material for: Dominance from the perspective of gene–gene and gene–chemical interactions
Source: Genetica. 2015 Nov 27;144:23–36. doi: 10.1007/s10709-015-9875-9 (PMC4748009; doi:10.1007/s10709-015-9875-9)

**Methods**

**Inferring genetic dominance phenotypes**

The Deutschbauer et al. study (2005; competitive fitness profiling of both homozygous and heterozygous deletion strains conducted in batch shake flasks) was the secondary source of HI and HS genes in our analysis for *S. cerevisiae*. The authors inferred HI genes in rich medium conditions from both fitness (heterozygous strains) and statistical criteria (fitness < 0.98 and at least one tag for a given gene being statistically significant, i.e., p-value < 0.05). Moreover, the authors manually curated the predicted datasets, filtering out genes considered by them to be false positives (e.g., genes with stronger fitness defect in the heterozygote (single allele knockout) than in the homozygote (knockout of both alleles) or cases with probable secondary mutations). The authors did not infer haplosufficient (HS) genes among heterozygous strains. However, they identified slow-growing homozygous strains using analogous fitness and statistical criteria as in the case of heterozygous strains. Thus, we defined the set of haplosufficient genes (HS) as those which are slow-growing or lethal (essential genes) in double knockouts (homozygous strains) and were not found to be HI genes (lack of significant growth defect in heterozygous strains).

Kim et al. (2010) reanalyzed the Deutschbauer et al. datasets for both heterozygous and homozygous strains. They made an effort to filter out non-reproducible data (by selecting only genes with average fitness < 0.97 and fitness defect in both pools < 0.02). We used the result of Kim et al. reanalysis as our third dataset of HI (slow-growing homozygous strains) and HS genes (slow-growing or lethal homozygous strains).

**Results**

**The datasets obtained from the Deutschbauer et al. study and Kim et al. reanalysis of this dataset do not support the findings obtained with the Pir et al. dataset**

We repeated the GI analyses with the HI and HS genes identified using the Deutschbauer et al. data. Surprisingly, we did not observe the trends that were clearly visible with the Pir et al. dataset, i.e., HI genes having significantly higher GI degree in comparison to HS genes (Fig. A1-A3). Moreover, in the case of negative binomial regression models, dominance was not found to be correlated with the GI degree (in both positive and negative GI networks from the Costanzo study and BioGRID; see Fig. A4).

We also repeated the chemogenetic analyses. The results were ambiguous. We found HI genes to have significantly more GCIs than recessive genes in heterozygous deletion strains and opposite (although insignificant) pattern in homozygous deletion strains (Fig. A5). We did not observe a correlation between dominance and the level of chemical perturbations in both homozygous and heterozygous deletion strains (after taking into account other dependent variables: fitness, gene pleiotropy, variation in gene expression and level of gene expression; see Fig. A6).

Interestingly, the level of chemical perturbations of both heterozygous and homozygous deletion strains was found to correlate significantly with the expression level, unlike the case for the Pir et al. dataset.

Moreover, the expression level was the only significant (dependent) variable in the regression model of heterozygote sensitivity to chemical perturbations, while, in the case of homozygous deletion strains, the level of chemical perturbation was found to correlate also with fitness defect and variation in gene expression.

We obtained analogous results with the Kim et al. datasets of HI and HS genes (comprising results of the reanalysis of Deutschbauer et al. data; results not shown).

**Discussion**

**The Deutschbauer et al. dataset represents a subset of HI genes enriched in translation-related gene and depleted in regulators of gene expression**

Deutschbauer *et al.* (2005) was the first group to analyze haploinsufficiency in *S. cerevisiae*. Thus, we repeated our key chemogenetic and GI network analyses with datasets from this study. Unfortunately, the results based on the Deutschbauer et al. datasets are not in agreement with the results observed for the Pir et al. dataset. We analyzed the Deutschbauer et al. datasets thoroughly and speculate that the observed disagreement is connected with the data quality (mainly due to the chosen culture type - batch culture), which resulted in a robust subset of (often translation-related) HI genes but probably not one representative for most HI genes. Our assumption is strongly supported by the note made by the authors on a supplementary page:

*“We recognize that all genomic level studies suffer from an inability to verify all measurements, therefore, generalizations must be made. Consequently, some of the strains with apparent fitness defects not indicated as "slow growth" are certain to reflect "real" biology, not an artifact. Therefore, while our lists of "slow growth" genes are robust (due to stringent selection criteria); they are probably not completely comprehensive.”*

In more detail, the authors applied a conservative procedure to restrict the analysis to highly confident HI genes. However, this procedure led to the enrichment of HI genes with strong fitness defects (fitness defect < 0.98) and, in some cases, with high fitness variation (genes with significant fitness decrease in at least one batch were also defined as HI genes). Most of them are essential genes (85%), often translation-related (ribosomal genes, gene participating in ribosomal biogenesis, gene of pre-ribosome) or encoding subunits of other macromolecular complexes (CCT folding chaperone, the exosome, the core subunit of RNA polymerase II; Deutschbauer et al. 2005). In contrast, the non-ribosomal HI set of genes in the Pir et al. (2010) study was enriched in genes with a small fitness defect but, at the same time, with very small fitness variation.

We confirmed our assumptions with the Gene Ontology analysis. Genes that are gene expression regulators (especially of the transcription process), which are enriched among non-ribosomal HI genes in the Pir et al. dataset, were found to be overrepresented among genes excluded from the Deutschbauer et al.(2005) study (because of data quality issues and applied methodology; see Online Resource 5).

In summary, some key analyses conducted with the Deutschbauer et al.(2005) datasets should be reproduced with the Pir et al. (2010) study, which seems to be genome-wide representative of HI and HS genes under rich medium conditions in the case of *S. cerevisiae*.

**References**

Deutschbauer AM et al. (2005) Mechanisms of haploinsufficiency revealed by genome-wide profiling in yeast Genetics 169:1915-1925 doi:genetics.104.036871

Kim DU et al. (2010) Analysis of a genome-wide set of gene deletions in the fission yeast Schizosaccharomyces pombe Nat Biotechnol 28:617-623 doi:10.1038/nbt.1628

Pir P, Gutteridge A, Wu J, Rash B, Kell DB, Zhang N, Oliver SG (2012) The genetic control of growth rate: a systems biology study in yeast BMC Syst Biol 6:4 doi:10.1186/1752-0509-6-4

Figures

**Fig. A1** GI degree (positive in the first column, negative in the second) observed for dominant haploinsufficient (in orange), recessive (in blue) and ribosomal (in green) genes in *S. cerevisiae*. Merged high-throughput studies from BioGRID and single high-throughput study from Costanzo were used. HI and HS sets were inferred from Deutschbauer et al. study. Haploinsufficient genes have (insignificantly) more GIs than recessive genes in Costanzo network while having less GIs in BioGRID-based networks. Ribosomal genes are depleted in GIs. Means are shown and error bars represent one standard deviation of the mean over 10000 bootstrapped samples of the distribution. Two-sample permutation test (two sided, p-values are shown above the error bars) was used to evaluate the difference between selected sets of genes. Number of genes in selected sets is shown in brackets. Horizontal dotted line represents the genome average. Abbreviations: HI – non-ribosomal haploinsufficient genes, HS – haplosufficient (recessive) genes, RIB – ribosomal genes


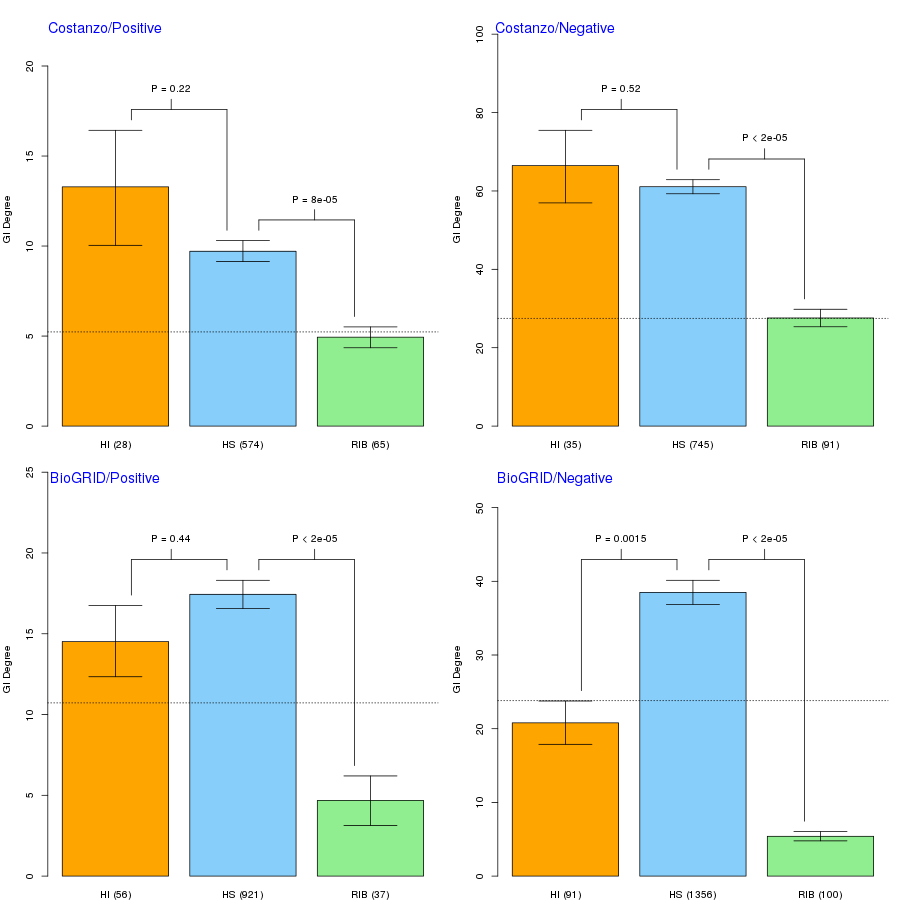


**Fig. A2** Distribution of selected properties (known to be correlated with GI degree) among three groups of S .cerevisiae genes: haploinsufficient genes (HI; in orange), haplosufficient genes (HS; recessive; in blue) and ribosomal genes (in green) inferred from Deutschbauer et al. study. Similarly as in case of HI genes inferred from Pir et al. study, HI genes (comparing to HS genes) are more important genes (stronger single fitness defect), more evolutionarily constrained ( higher evolutionary conservation) and more pleiotropic (i.e. participate in more functions in the cell as indicated by: higher number of Gene Ontology terms (multifunctionality), higher number of protein-protein interactions). However, opposite to Oliver’s dataset, HI genes (in comparison to HS genes) have higher variation in genes expression. Ribosomal genes (in comparison to HS genes and genome average), similarly as HI genes are more important genes and have higher gene expression (one order of magnitude difference). However, (opposite to HI genes) ribosomal genes are less pleiotropic. Ribosomal genes were filtered out from both HI and HS groups. Means are shown and error bars represent one standard deviation of the mean over 10000 bootstrapped samples of the distribution. Two-sample permutation test (two sided, p-values are shown above the error bars) was used to evaluate the difference between selected sets of genes. Number of genes in selected sets is shown in brackets. Horizontal dotted line represents the genome average. Abbreviations: HI – non-ribosomal haploinsufficient genes, HS – non ribosomal haplosufficient (recessive) genes, RIB – ribosomal genes

**
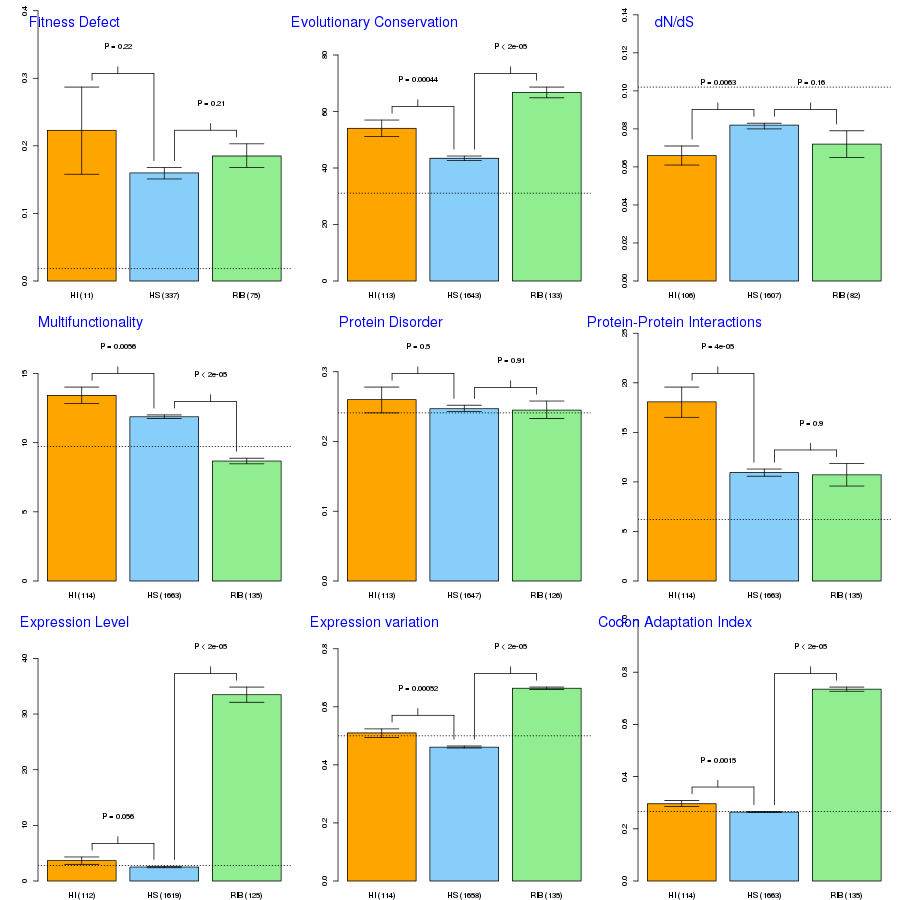
**

**Fig. A3** Comparison of distribution of gene expression variation (known to be negatively correlated with GI degree) among three groups of genes: haploinsufficient genes (HI; in orange), haplosufficient genes (HS; recessive; in blue) and ribosomal genes (in green) inferred from Deutschbauer et al. study. Opposite to dataset inferred from Pir et al. study HI genes have ambiguous pattern of gene expression variation Ribosomal genes were filtered out from both HI and HS groups. Means are shown and error bars represent one standard deviation of the mean over 10000 bootstrapped samples of the distribution. Two-sample permutation test (two sided, p-values are shown above the error bars) was used to evaluate the difference between selected sets of genes. Number of genes in selected sets is shown in brackets. Horizontal dotted line represents the genome average. Abbreviations: HI – non-ribosomal haploinsufficient genes, HS – non ribosomal haplosufficient (recessive) genes, RIB – ribosomal genes, STN – stochasticity, RES – responsiveness, TRV – trans variability, MUV – mutational variance, ISV – interstrain variation, MUV – mutational variance


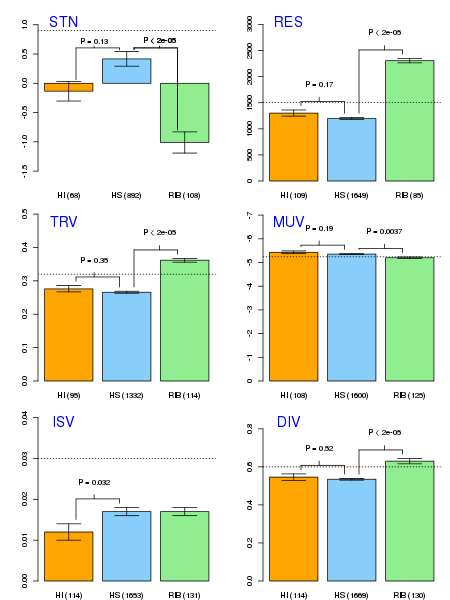


**Fig. A4** Comparison of effects of selected properties: evolutionary constraints (as single mutant fitness – in blue), multifunctionality (in red), genetic dominance (in beige), variation in gene-expression (in grey) and level of gene expression (in violet) on GI degree. Negative binomial regression was carried out for each GI network as a function of selected properties. Significant correlation between dominance and GI degree was not observed in any case, after taking into account confounding factors (especially single mutant fitness and multifunctionality). The statistical significance of regression is shown by -log10 (p-value) on the y axis. The threshold of statistical significance is 1.3 (-log10 of 0.05). Analysis was conducted for *S. cerevisiae* HI and HS genes identified in Deutschbauer et al. study. Numbers of genes analyzed in each GIs network are shown in brackets. Abbreviations: BNEG: negative GIs from BioGRID; CNEG: negative GIs from Costanzo study; BPOS: positive GIs from BioGRID; CPOS: negative GIs from Costanzo study


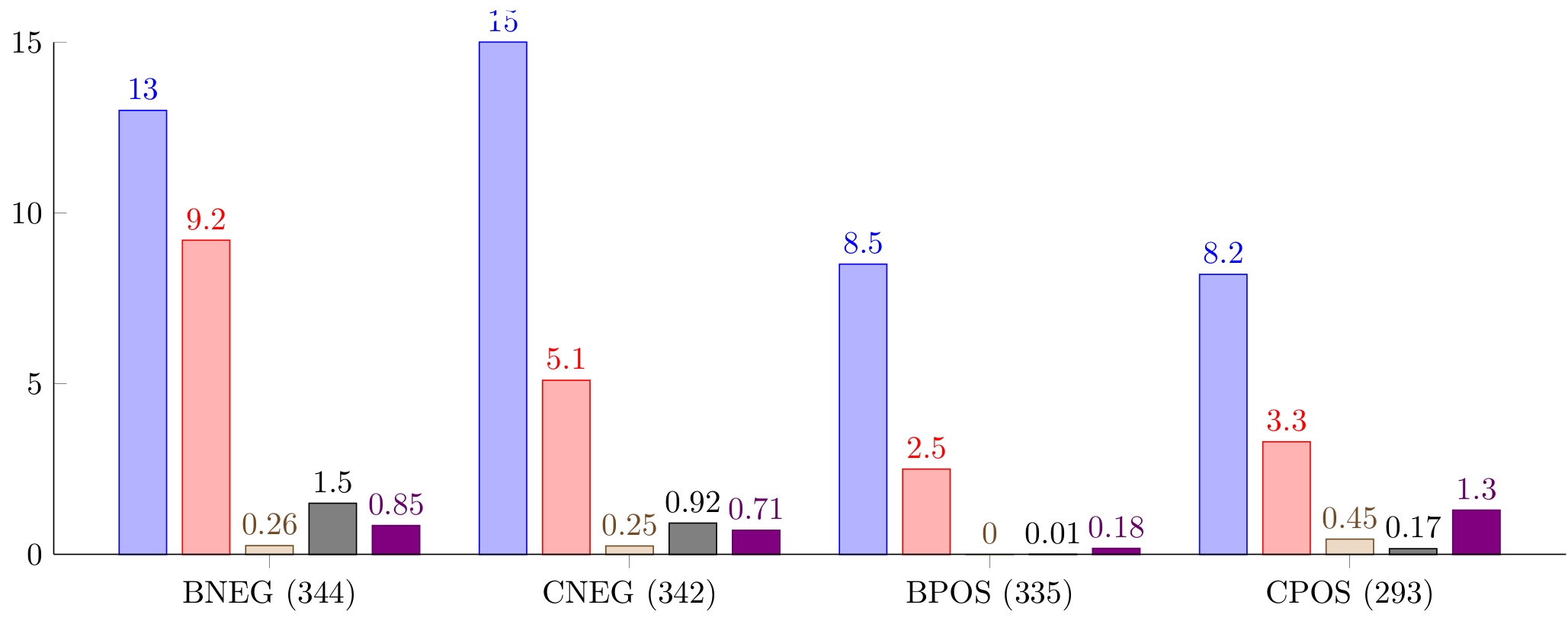


**Fig. A5** Degree of gene-chemical interactions (for heterozygous deletion mutants in the first column and homozygous deletion mutants in in the second) observed for dominant haploinsufficient (in orange), recessive (in blue) and ribosomal (in green) genes in *S. cerevisiae*. Single high-throughput study by Hillenmayer et al. was used. HI and HS sets were inferred from Deutschbauer et al. study. Haploinsufficient genes have significantly more gene-chemical interactions than recessive ones in case of heterozygous deletion knockouts and (insignificantly) less gene-chemical interactions in case of homozygous deletion knockouts. Ribosomal genes are depleted in gene-chemical interactions. Means are shown and error bars represent one standard deviation of the mean over 10000 bootstrapped samples of the distribution. Two-sample permutation test (two sided, p-values are shown above the error bars) was used to evaluate the difference between selected sets of genes. Number of genes in selected sets is shown in brackets. Horizontal dotted line represents the genome average. Abbreviations: HI – non-ribosomal haploinsufficient genes, HS – haplosufficient (recessive) genes, RIB – ribosomal genes, Chemo: Het: heterozygous chemogenetic network; Chemo Hom: homozygous chemogenetic network


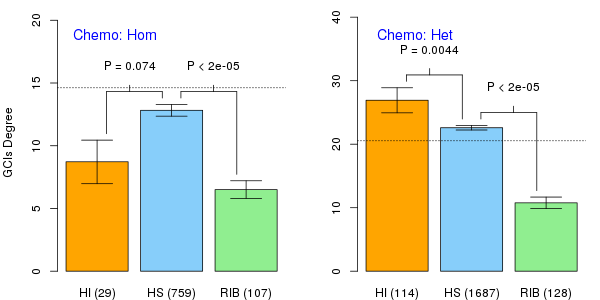


**Fig. A6** Comparison of effects of selected properties: evolutionary constraints (as single mutant fitness – in blue), multifunctionality (in red), genetic dominance (in beige), variation in gene-expression (in grey) and level of gene expression (in violet) on GCIs degree. Negative binomial regression was carried out for each chemogenetic network (build upon collection of homozygous and heterozygous deletion mutants) as a function of selected properties. In both, homozygous and heterozygous chemogenetic networks dominance does not significantly affect GI degree, after taking into account confounding factors (single mutant fitness, multifunctionality, variation in gene expression and gene expression level). The statistical significance of regression is shown by -log10 (p-value) on the y axis. The threshold of statistical significance is 1.3 (-log10 of 0.05). Analysis was conducted for *S. cerevisiae* HI and HS genes identified in Deutschbauer et al. study. Numbers of genes analyzed in each GCIs network are shown in brackets. Abbreviations: Chemo: Het: heterozygous chemogenetic network; Chemo: Hom homozygous chemogenetic network


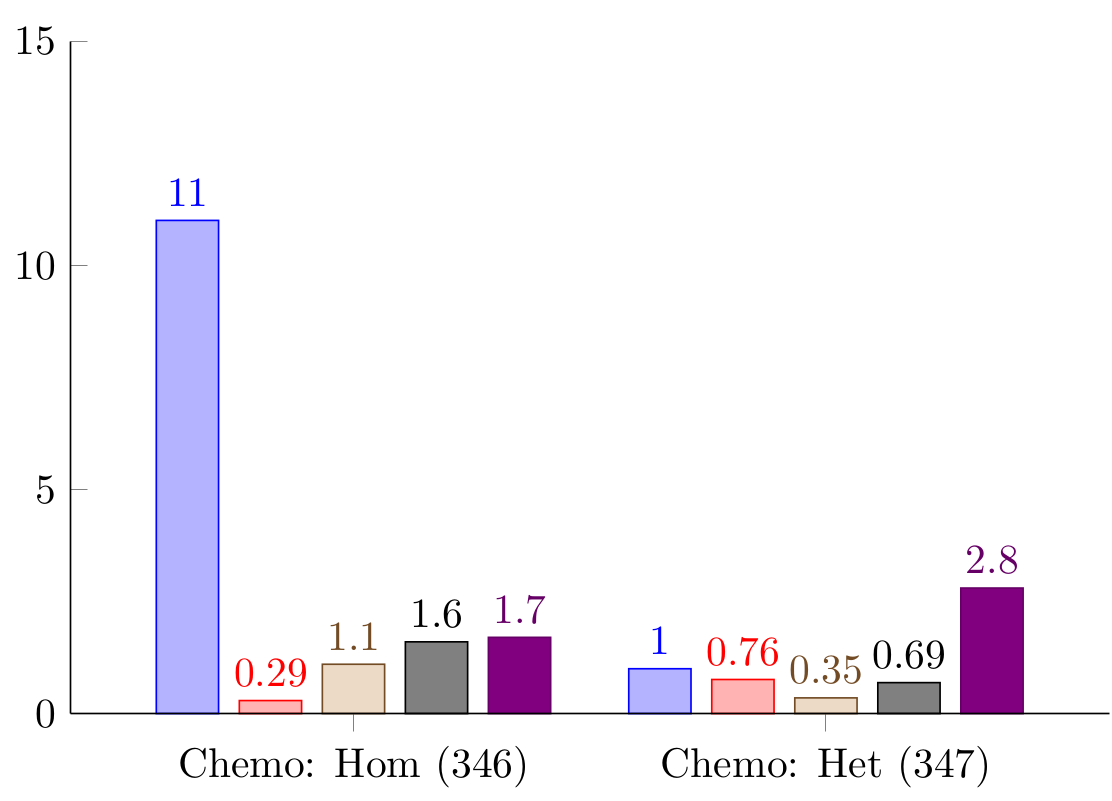

Supplement: Supplementary file 4 — Additional methods and results for the analyses conducted for S. cerevisiae with Deutschbauer et al. dataset (DOC 277 kb) [file 10709_2015_9875_MOESM4_ESM.doc]
