# Supplementary material for: Dominance from the perspective of gene–gene and gene–chemical interactions
Source: Genetica. 2015 Nov 27;144:23–36. doi: 10.1007/s10709-015-9875-9 (PMC4748009; doi:10.1007/s10709-015-9875-9)

**Methods**

**Inferring genetic dominance phenotypes**

We made use of hybridization data (Affymetrix CEL files) from the Steinmetz et al. study (2002) to predict HI and HS genes similarly as in the case of (Deutschbauer et al. 2005). We restricted our analysis to homozygous and heterozygous strains from YPD media.

We used the scripts made available by Pierce et al. (2006; web supplementary materials) to preprocess CEL files (removal of tags with same distribution as background, raw_file_maker.pl script) and to normalize data (we slightly modified the normalize_data.pl script to perform same normalization as Deutschbauer et al. did: to standardize to mean intensity across all tags in the corresponding pool).

As in the case of Deutschbauer et al., for each tag we conducted a multiple linear regression with the same dependent variables (time effects, replicated series effects and series-time interactions). With an in-house R script, we made use of the glm function (Venables and Ripley 2002) ,using the default method for robust model fitting (IWLS). As in the case of Deutschbauer et al. we added 1 to the regression slope to obtain the relative fitness of a given tag. Then, we calculated the mean fitness value of a given deletion strain in a given pool by averaging the individual fitness tag values of the deletion strain.

In the Deutschbauer et al. Study, two pools were conducted for each type of deletion (homozygous, heterozygous). In each pool, there were six replicates for five time series. In the case of Steinmetz, there was only one pool conducted for each type of deletion (homozygous, heterozygous), with two replicates for five time series. In the case of Deutschbauer et al., a given gene was predicted to be HI if it was in both heterozygous pools: average fitness value for the gene was < 0.98 (1) and at least one tag for a given gene was found to be statistically significant, i.e., p-value < 0.05 (2). As there was only a single heterozygous pool in Steinmetz et al., we had to modify the procedure for HI prediction (average fitness value for given gene in single heterozygous pool < 0.98 (1) and at least one tag for a given gene found to be statistically significant, i.e., p-value < 0.05 in single heterozygous pool). For haplosufficient (HS) gene prediction, we also changed the procedure (compared to the Deutschbauer dataset) in an analogous way. Note that such changes make the datasets of HI and HS genes predicted with Steinmetz et al. data more prone to false positives.

**Results**

**The datasets obtained from the Steinmetz et al. study do not support the findings obtained with the Pir et al. dataset**

We predicted 186 non-ribosomal HI genes, 55 ribosomal HI genes and 1581 HS genes ( 602 predicted in the study and 979 essential genes which were not found to be HI). See Online Resource 7 for the list of predicted HI and HS genes).

We repeated the GI analyses with the HI and HS genes identified using the Steinmetz et al. data. We observed ambiguous (often opposite) trends compared with the results obtained with Pir et al. Importantly, we found HS genes to have a higher negative GI degree in comparison with HI genes (Fig. A1), stronger fitness defect and lower gene expression variation (Fig. A2). Moreover, in the case of negative binomial regression models, dominance was found to correlate negatively with GI degree (in negative GI networks from the Costanzo’s study and BioGRID; see Fig. A3).

We also repeated the chemogenetic analyses. The results were also ambiguous. We found HI genes to have significantly more GCIs than recessive genes in heterozygous deletion strains and the opposite significant pattern in homozygous deletion strains (Fig. A4). Both observations also stayed valid after taking into account other dependent variables: fitness, gene pleiotropy, variation in gene expression and level of gene expression; see Fig. A5).

**Discussion**

**The Steinmetz et al. dataset comprises a subset of HI genes enriched in translation-related gene and HS genes enriched in regulators of gene expression**

In the case of Deutschbauer et al. (2005), we found that the genes excluded from their analysis are often regulators of gene expression process, especially transcription. Here, in case of Steinmetz et al., we found this functional class of genes to be misclassified as HS genes (see Online Resource 9) which explains why the results from Steinmetz et al. apparently contradict the results from the Pir et al. dataset.

In general, haploinsufficiency studies in *S. cerevisiae* designed in batch cultures (Deutschbauer et al., Steinmetz et al.) are biased due to the unreliable measurement of fitness defects for deletion mutants of genes being regulators of gene expression. This is not the case for the Olivier et al. study, which was conducted in continuous culture (chemostat and turbidostat).

**References**

Deutschbauer AM, Jaramillo DF, Proctor M, et al (2005) Mechanisms of haploinsufficiency revealed by genome-wide profiling in yeast. Genetics 169:1915–25. doi: 10.1534/genetics.104.036871

Hillenmeyer ME, Ericson E, Davis RW, et al (2010) Systematic analysis of genome-wide fitness data in yeast reveals novel gene function and drug action. Genome Biol 11:R30. doi: 10.1186/gb-2010-11-3-r30

Pierce SE, Fung EL, Jaramillo DF, et al (2006) A unique and universal molecular barcode array. Nat Methods 3:601–603. doi: 10.1038/nmeth905

Steinmetz LM, Scharfe C, Deutschbauer AM, et al (2002) Systematic screen for human disease genes in yeast. Nat Genet 31:400–404. doi: 10.1038/ng929

Venables WN, Ripley BD (2002) Modern applied statistics with S-Plus. Springer, New York [etc.]

Figures

**Fig. A1** GI degree (positive in the first column, negative in the second) observed for dominant haploinsufficient (in orange), recessive (in blue) and ribosomal (in green) genes in *S.cerevisiae.* Merged high-throughput studies from BioGRID and single high-throughput study from Costanzo were used. HI and HS sets were inferred from Steinmetz et al. study. Means are shown and error bars represent one standard deviation of the mean over 10000 bootstrapped samples of the distribution. Two-sample permutation test (two sided, p-values are shown above the error bars) was used to evaluate the difference between selected sets of genes. Number of genes in selected sets is shown in brackets. Horizontal dotted line represents the genome average. Abbreviations: HI – non-ribosomal haploinsufficient genes, HS – haplosufficient (recessive) genes, RIB – ribosomal genes


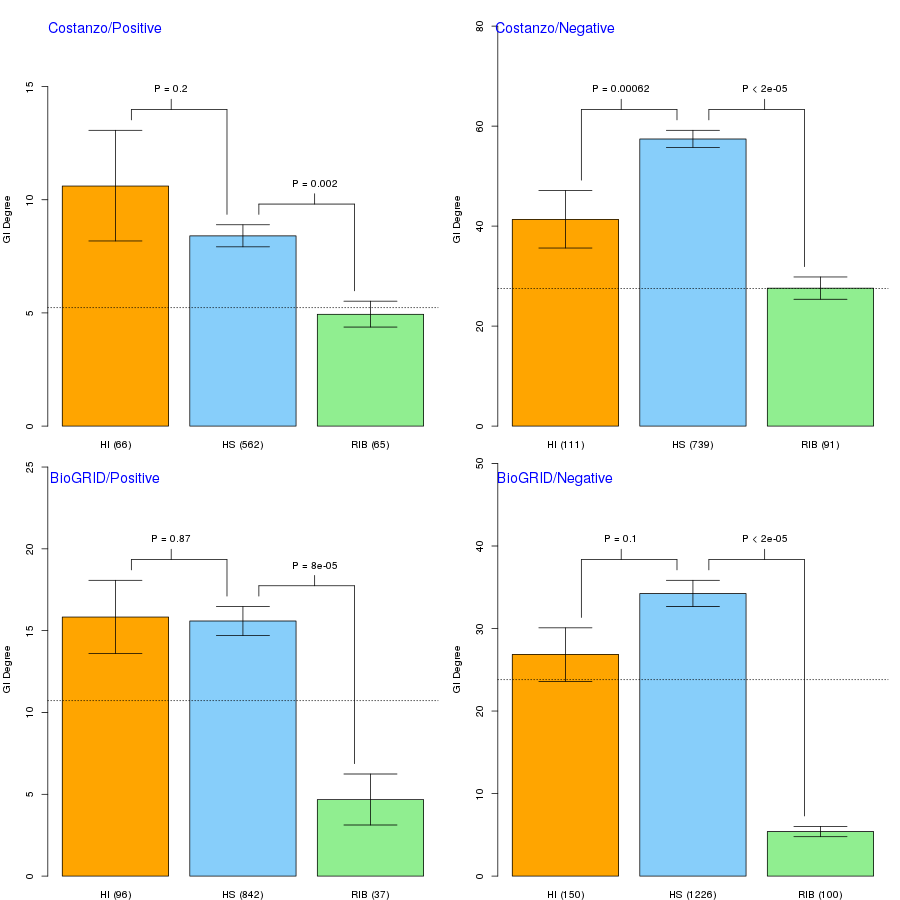


**Fig. A2** Distribution of selected properties (known to be correlated with GI degree) among three groups of S .cerevisiae genes: haploinsufficient genes (HI; in orange), haplosufficient genes (HS; recessive; in blue) and ribosomal genes (in green) inferred from Steinmetz et al. study. Opposite to HI genes inferred from Pir et al. study, HI genes (comparing to HS genes) are more less important genes (weaker single fitness defect),have higher variation in genes expression. Ribosomal genes (in comparison to HS genes and genome average), similarly as HI genes are more important genes and have higher gene expression (one order of magnitude difference). However, (opposite to HI genes) ribosomal genes are less pleiotropic. Ribosomal genes were filtered out from both HI and HS groups. Means are shown and error bars represent one standard deviation of the mean over 10000 bootstrapped samples of the distribution. Two-sample permutation test (two sided, p-values are shown above the error bars) was used to evaluate the difference between selected sets of genes. Number of genes in selected sets is shown in brackets. Horizontal dotted line represents the genome average. Abbreviations: HI – non-ribosomal haploinsufficient genes, HS – non ribosomal haplosufficient (recessive) genes, RIB – ribosomal genes

**
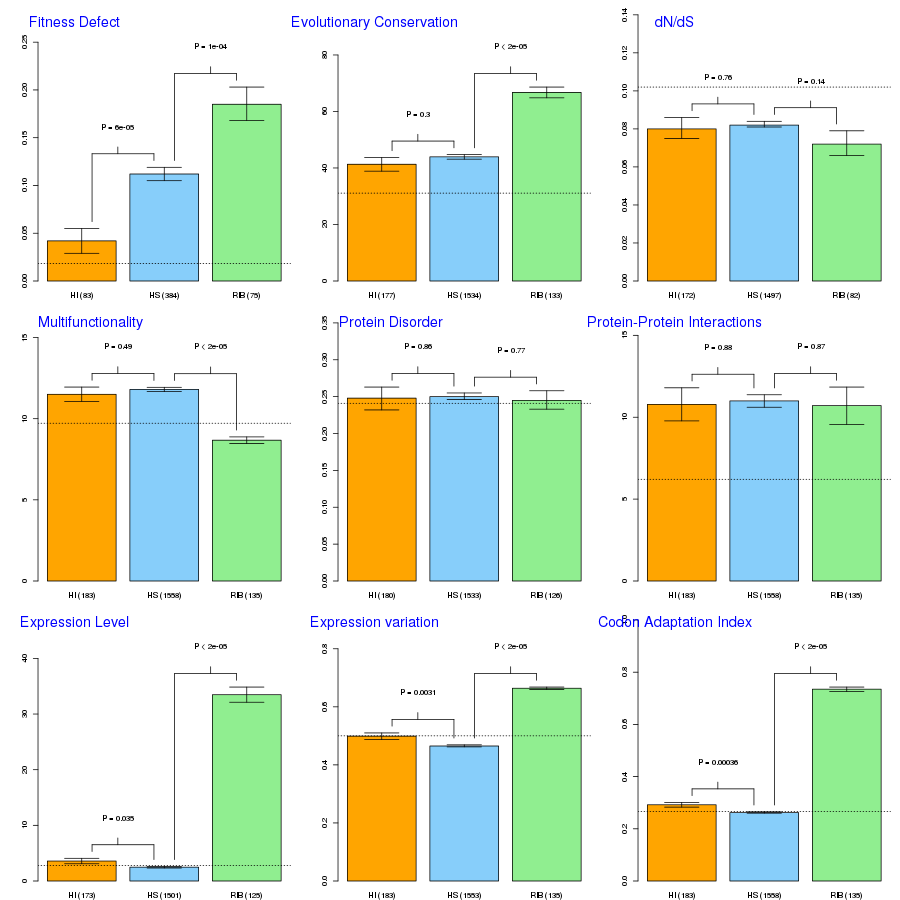
**

**Fig. A3** Comparison of effects of selected properties: evolutionary constraints (as single mutant fitness – in blue), multifunctionality (in red), genetic dominance (in beige), variation in gene-expression (in grey) and level of gene expression (in violet) on GI degree. Negative binomial regression was carried out for each GI network as a function of selected properties. Significant negative correlation between dominance and GI degree was observed in case of networks of negative GIs, after taking into account confounding factors (especially single mutant fitness and multifunctionality). The statistical significance of regression is shown by -log10 (p-value) on the y axis. The threshold of statistical significance is 1.3 (-log10 of 0.05). Analysis was conducted for *S. cerevisiae* HI and HS genes identified in Steinmetz et al. study. Numbers of genes analyzed in each GIs network are shown in brackets. Abbreviations: BNEG: negative GIs from BioGRID; CNEG: negative GIs from Costanzo study; BPOS: positive GIs from BioGRID; CPOS: negative GIs from Costanzo study


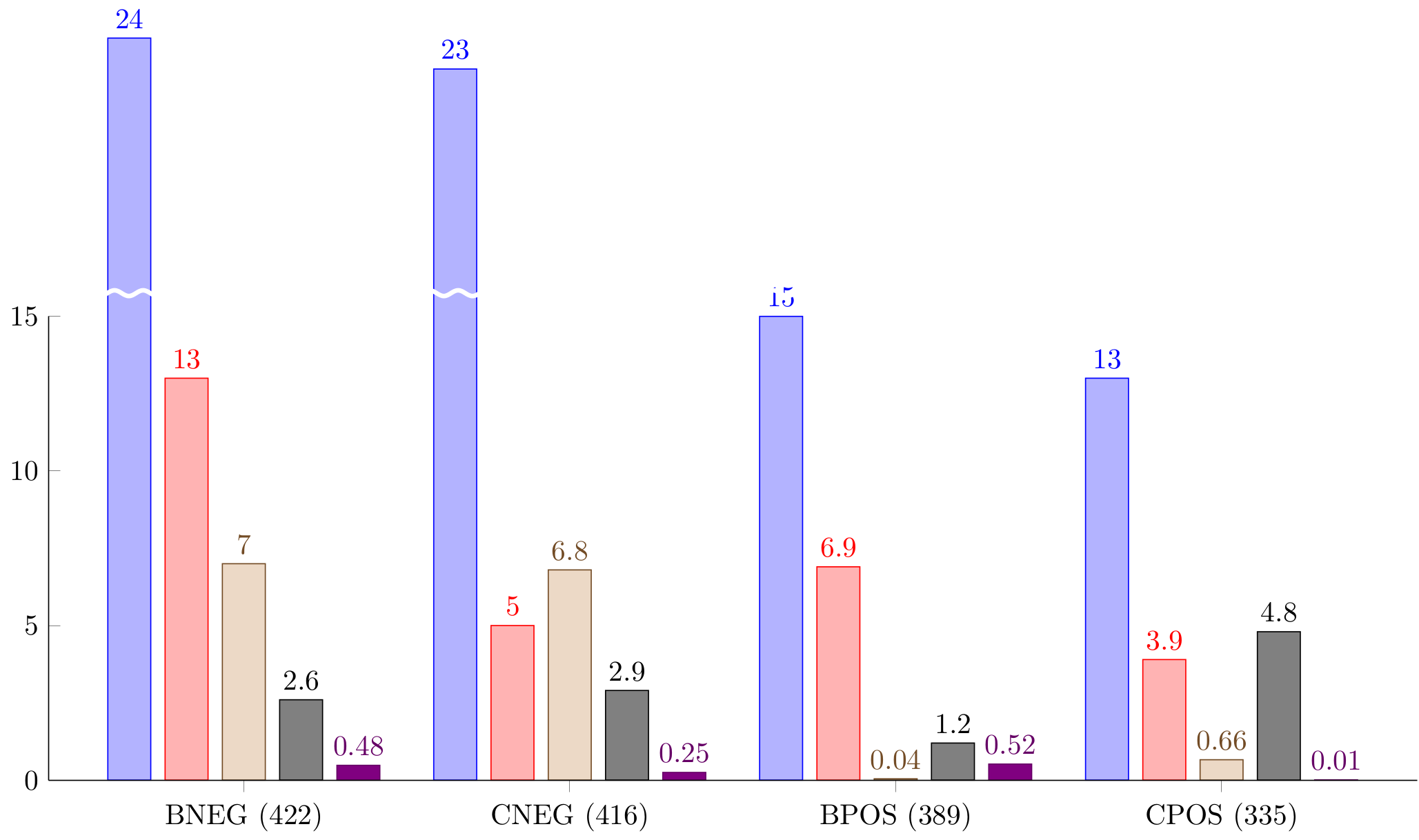


**Fig. A4** Degree of gene-chemical interactions (for heterozygous deletion mutants in the first column and homozygous deletion mutants in in the second) observed for dominant haploinsufficient (in orange), recessive (in blue) and ribosomal (in green) genes in *S. cerevisiae*. Single high-throughput study by Hillenmayer et al. was used. HI and HS sets were inferred from Steinmetz et al. study. Haploinsufficient genes have significantly more gene-chemical interactions than recessive ones in case of heterozygous deletion knockouts and significantly less gene-chemical interactions in case of homozygous deletion knockouts. Ribosomal genes are depleted in gene-chemical interactions. Means are shown and error bars represent one standard deviation of the mean over 10000 bootstrapped samples of the distribution. Two-sample permutation test (two sided, p-values are shown above the error bars) was used to evaluate the difference between selected sets of genes. Number of genes in selected sets is shown in brackets. Horizontal dotted line represents the genome average. Abbreviations: HI – non-ribosomal haploinsufficient genes, HS – haplosufficient (recessive) genes, RIB – ribosomal genes, Chemo: Het: heterozygous chemogenetic network; Chemo Hom: homozygous chemogenetic network


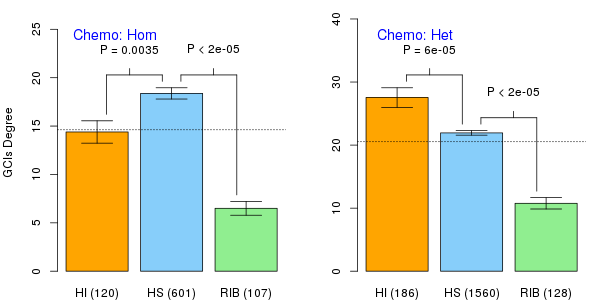


**Fig. A5** Comparison of effects of selected properties: evolutionary constraints (as single mutant fitness – in blue), multifunctionality (in red), genetic dominance (in beige), variation in gene-expression (in grey) and level of gene expression (in violet) on GCIs degree. Negative binomial regression was carried out for each chemogenetic network (build upon collection of homozygous and heterozygous deletion mutants) as a function of selected properties. In both, homozygous and heterozygous chemogenetic networks dominance does significantly (but near the 0.05 threshold) affect GI degree, after taking into account confounding factors (single mutant fitness, multifunctionality, variation in gene expression and gene expression level). The statistical significance of regression is shown by -log10 (p-value) on the y axis. The threshold of statistical significance is 1.3 (-log10 of 0.05). Analysis was conducted for *S. cerevisiae* HI and HS genes identified in Steinmetz et al. study. Numbers of genes analyzed in each GCIs network are shown in brackets. Abbreviations: Chemo: Het: heterozygous chemogenetic network; Chemo: Hom homozygous chemogenetic network


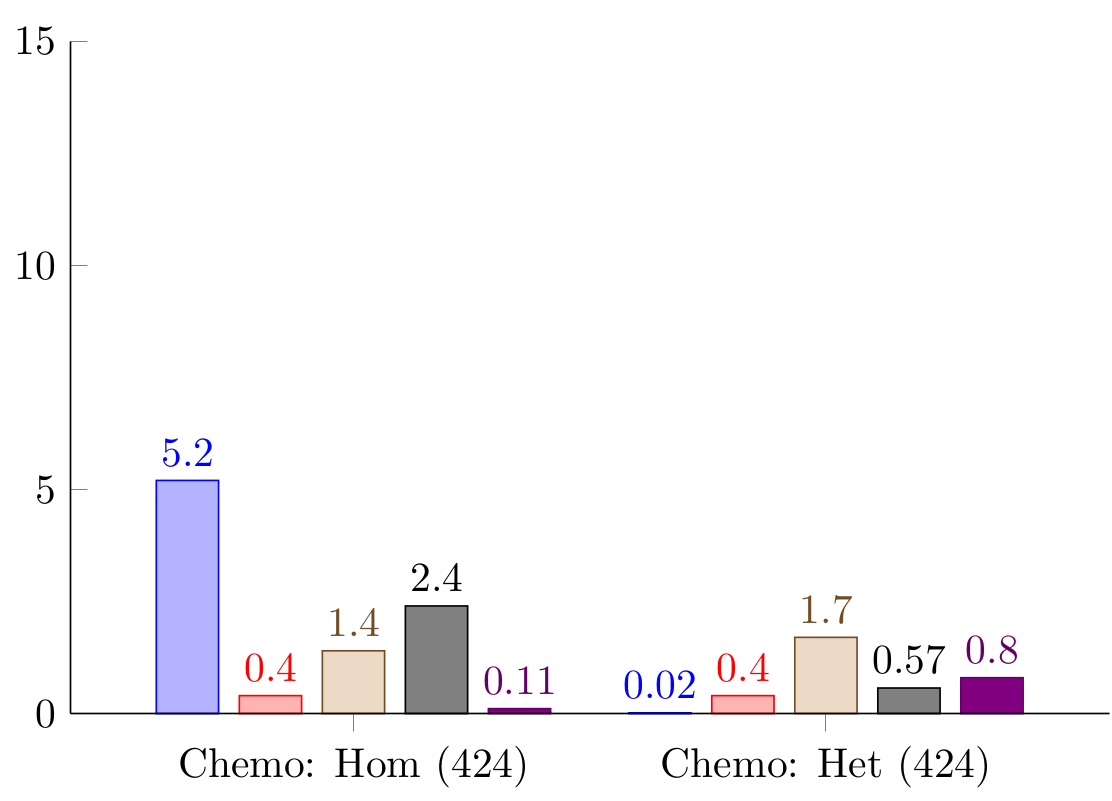

Supplement: Supplementary file 6 — Additional methods and results for the analyses conducted for S. cerevisiae with the Steinmetz et al. dataset (DOC 222 kb) [file 10709_2015_9875_MOESM6_ESM.doc]
